# Clonal selection of hematopoietic stem cells after gene therapy for sickle cell disease

**DOI:** 10.1038/s41591-023-02636-6

**Published:** 2023-11-16

**Authors:** Michael Spencer Chapman, Alyssa H. Cull, Marioara F. Ciuculescu, Erica B. Esrick, Emily Mitchell, Hyunchul Jung, Laura O’Neill, Kirsty Roberts, Margarete A. Fabre, Nicholas Williams, Jyoti Nangalia, Joanne Quinton, James M. Fox, Danilo Pellin, Julie Makani, Myriam Armant, David A. Williams, Peter J. Campbell, David G. Kent

**Affiliations:** 1 Wellcome Sanger Institute, Hinxton, UK; 2 Department of Haematology, Hammersmith Hospital, Imperial College Healthcare NHS Trust, London, UK; 3 Department of Haematology, Cambridge University Hospitals NHS Foundation Trust, Cambridge, UK; 4 York Biomedical Research Institute, University of York, York, UK; 5 Division of Hematology/Oncology, Boston Children’s Hospital, Boston, MA USA; 6 Department of Pediatric Oncology, Dana-Farber Cancer Institute, Boston, MA USA; 7 Harvard Medical School, Boston, MA USA; 8 Wellcome-Medical Research Council Cambridge Stem Cell Institute, Cambridge, UK; 9 Centre for Genomics Research, Discovery Sciences, BioPharmaceuticals R&D, AstraZeneca, Cambridge, UK; 10 Gene Therapy Program, Dana-Farber/Boston Children’s Cancer and Blood Disorders Center, Harvard Medical School, Boston, MA USA; 11 Muhimbili University of Health and Allied Sciences (MUHAS), Dar-es-Salaam, Tanzania; 12 SickleInAfrica Clinical Coordinating Center, MUHAS, Dar-es-Salaam, Tanzania; 13 Imperial College London, London, UK; 14 Harvard Stem Cell Institute, Cambridge, MA USA

**Keywords:** Stem-cell research, Haematological diseases

## Abstract

Gene therapy (GT) provides a potentially curative treatment option for patients with sickle cell disease (SCD); however, the occurrence of myeloid malignancies in GT clinical trials has prompted concern, with several postulated mechanisms. Here, we used whole-genome sequencing to track hematopoietic stem cells (HSCs) from six patients with SCD at pre- and post-GT time points to map the somatic mutation and clonal landscape of gene-modified and unmodified HSCs. Pre-GT, phylogenetic trees were highly polyclonal and mutation burdens per cell were elevated in some, but not all, patients. Post-GT, no clonal expansions were identified among gene-modified or unmodified cells; however, an increased frequency of potential driver mutations associated with myeloid neoplasms or clonal hematopoiesis (*DNMT3A*- and *EZH2*-mutated clones in particular) was observed in both genetically modified and unmodified cells, suggesting positive selection of mutant clones during GT. This work sheds light on HSC clonal dynamics and the mutational landscape after GT in SCD, highlighting the enhanced fitness of some HSCs harboring pre-existing driver mutations. Future studies should define the long-term fate of mutant clones, including any contribution to expansions associated with myeloid neoplasms.

## Abstract (web summary)

Analysis of hematopoietic stem cells from six individuals with sickle cell disease who had been treated with autologous gene therapy revealed positive selective pressure on cells containing mutations in genes associated with clonal hematopoiesis and hematological malignancies.

## Main

GT treatments for various diseases are becoming increasingly available to patients, with hundreds of clinical trials currently active in the United States alone^1^. Pioneering studies laid the groundwork for using GT to cure difficult-to-treat monogenic diseases such as X-linked severe combined immunodeficiency, adenosine deaminase-deficient severe combined immunodeficiency, leukodystrophies and other genetic disorders^2–10^. Early successes in this field were initially dampened by reports of patients who developed vector insertion-related leukemias directly linked to the viral platform used for transgene delivery^11–18^. Although insertional mutagenesis risk has been reduced by improved vector design^19^, the development of myelodysplastic syndrome (MDS) and acute myeloid leukemia (AML) at 3–5.5 years post-transplantation in 2 of 47 patients who had undergone GT for SCD^20–23^ has generated renewed concerns. In contrast to previously reported leukemogenesis events, the causative genetic lesions in these GT recipients do not seem to be linked to insertional mutagenesis. The factors promoting the development of these blood cancers therefore remain unknown. In these and other instances of GT-related malignancies, disease-specific or genetic factors may play a role. These adverse events have highlighted the need to understand pre- and post-GT genomic landscapes and stem cell dynamics. In this study, we used whole-genome sequencing (WGS) of individual hematopoietic stem and progenitor cells (HSPCs) to explore the genetic consequences of SCD and GT on the stem cell pool.

## Results

**Extended Data Fig. 1:**
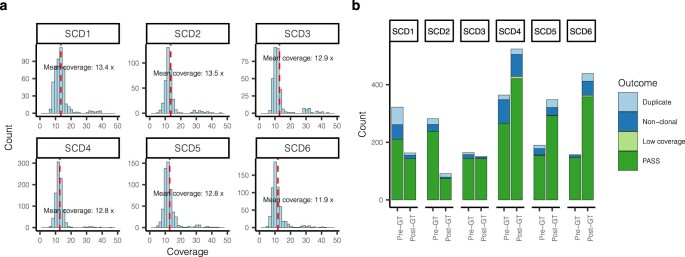
Sequencing coverage and colony outcomes. **a**, Histograms of sequencing coverage of all colonies that had >4× coverage, divided by individual. Mean coverage values per individual are indicated. **b**, Final outcomes of all colonies submitted for whole-genome sequencing, separated by individual and time point. Colonies with <4× sequencing coverage were excluded as insufficient coverage. Non-clonal and duplicate samples were identified as described in Methods.

There are a number of mechanisms by which the risk of leukemic transformation in SCD GT trials could be increased: (1) an elevated mutation rate due to SCD itself; (2) mutations resulting from ex vivo manipulation and transplantation of HSCs, including insertional mutagenesis; (3) mutations in any surviving residual HSC fraction due to conditioning chemotherapy unrelated to vector insertions; and (4) positive selective pressure on HSCs containing pre-existing driver mutations. We explored each of these possibilities using our recently developed approach that permits the study of human HSC clonal dynamics and relatedness using somatic mutations as unique molecular barcodes^24^. Our study cohort consisted of six individuals aged 7–26 years old who had been diagnosed with severe SCD (HbSS or HbSβ^0^-thalassemia) and had undergone GT (Table 1). The clinical trial (NCT03282656) utilized plerixafor-mobilized CD34^+^ peripheral blood cells transduced with a short hairpin RNA embedded in a microRNA (shmiR) that induces knockdown of *BCL11A*, leading to the de-repression of γ-globin expression and induction of fetal hemoglobin^25^. DNA was extracted from HSPC-derived colonies grown in MethoCult medium from fresh or viably frozen samples and WGS was performed at an average sequencing depth of 12.7× on 315–888 colonies per individual (Extended Data Fig. 1a). For all patients, colonies were derived from samples collected at both pre- and post-GT time points (Extended Data Fig. 1b and Supplementary Table 1). Across the 2,592 whole genomes, we identified 843,305 independently acquired single-nucleotide variants (SNVs) and 20,228 insertions and deletions (indels).

**Table 1:** Patient characteristics and colony sequencing information

Patient ID	Age in years, sex	Genotype	CD34^+^ cells transduced	Infused CD34^+^ cell dose (10^6^ cells per kg)	Sequencing depth	No. colonies sequenced
SCD1	7, male	β^S^/β^S^	62.0%	4.86	13.3×	354
SCD2	13, female	β^S^/β^S^	81.7%	3.55	13.5×	312
SCD3	16, female	β^S^/β^0^	100%	8.26	13.0×	287
SCD4	20, male	β^S^/β^S^	95.8%	5.07	12.9×	687
SCD5	24, male	β^S^/β^S^	95.5%	5.15	12.9×	447
SCD6	26, male	β^S^/β^S^	98.6%	6.70	11.9×	505

### Somatic mutations in patients with SCD

**Fig. 1:**
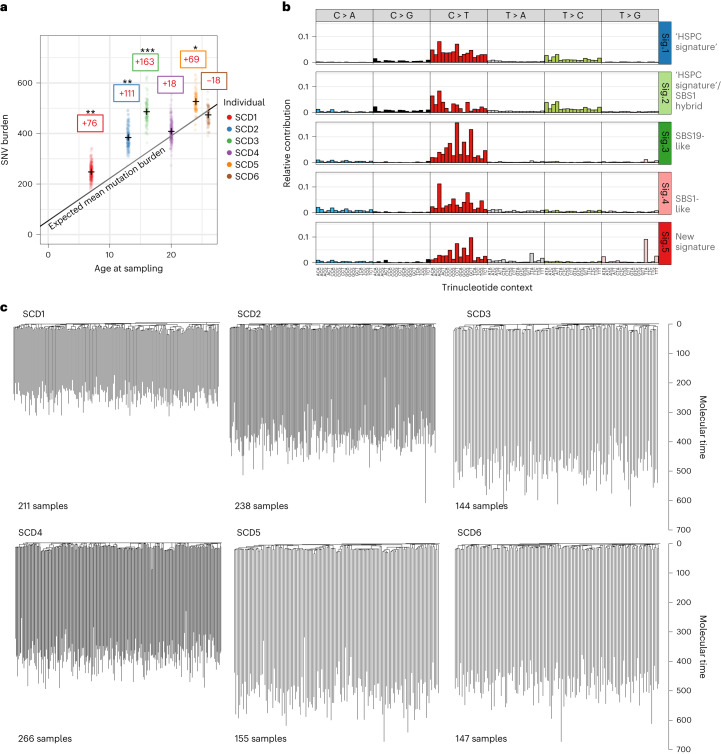
Landscape of somatic mutations in SCD. **a**, Dot-plot showing the number of mutations per HSPC for each patient plotted against the patient age at the time of sampling. SNV mutation burdens of individual HSPC colonies from before GT, with correction for coverage, are displayed per patient. Mean mutation burdens per individual are indicated by a cross. The black line indicates the expected mean mutation burden by age from a previous study looking at hematopoietically healthy individuals^28^. The average total number of mutations per HSPC above (+)/below (−) the expected value is indicated in the colored boxes. The mutation burdens for each patient were individually tested against the reference mutation set using a linear mixed-effects model with ‘age’ and ‘patient/reference status’ as fixed effects, and ‘individual’ as a random effect, to see if the ‘patient/reference status’ term was significant (*P < 0.05, **P < 0.01, ***P < 0.001). Exact *P* values for SCD1 to SCD6 were 9.5 × 10^−3^, 1.1 × 10^−3^, 9.7 × 10^−5^, 0.41, 1.0 × 10^−2^ and 0.54, respectively. **b**, Mutational signature analysis reflecting the underlying mutational processes that have been active within sequenced HSPCs. Signatures incorporate the base substitution types in the context of the bases immediately 5′ and 3′ to the mutated bases. Interpretation of each signature, by comparison with known signatures, is shown to the right of each profile. The contributions of each signature to each sample are shown in Extended Data Fig. 2c. Sig., signature. **c**, Phylogenies showing relatedness of the pre-GT colonies from each individual. Branches are scaled by the number of mutations allocated to that branch and corrected for sequencing depth such that branch lengths reflect the number of mutations acquired in that ancestral lineage. Given the fairly constant rate of mutation acquisition, this is a surrogate for time passed in that lineage and is termed ‘molecular time’.

**Extended Data Fig. 2:**
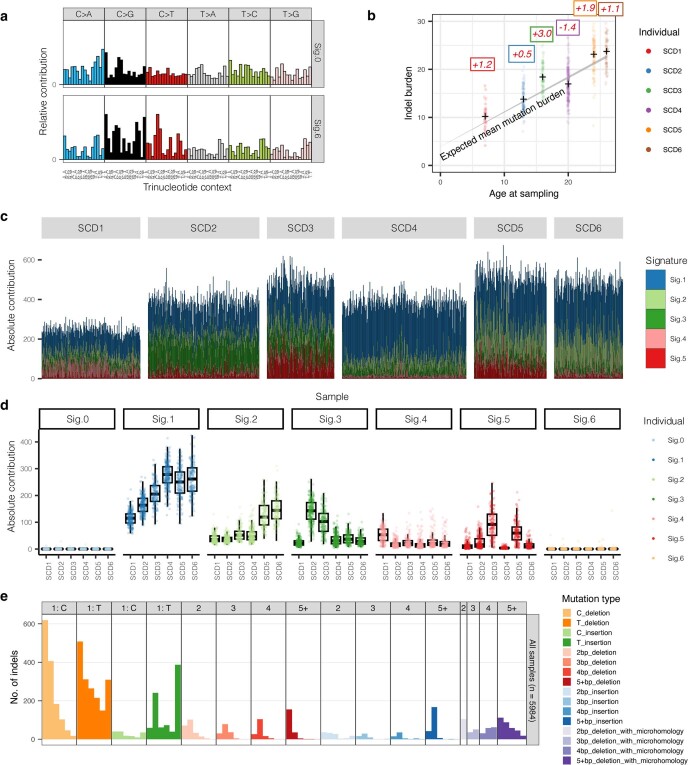
Mutation burdens and signatures prior to gene therapy. **a**, Extracted mutational signatures that were deemed likely to be due to artefactual / *in vitro*-acquired mutations, generally accounting for small numbers of mutations in each sample. **b**, As per Fig. 1a, but for indels. **c**, Barplot showing contributions of each mutational signature to the mutation burden of individual samples. Each vertical line represents the mutations in a sample, with the color indicating the absolute contribution of each signature to those mutations. **d**, Absolute mutation contributions of each signature per sample by individual (n=2,593 colonies total). The box-and-whisker plots show the distribution of absolute mutation signature contributions per colony within each individual, with the boxes indicating median and interquartile range. The upper whisker extends from the hinge to the largest value no further than 1.5 * IQR from the hinge and the lower whisker extends from the hinge to the smallest value at most 1.5 * IQR of the hinge. The overlaid points are the jittered observed signature contributions to individual colonies. **e**, Indel mutational signature profile across all samples. Sig. = signature.

**Extended Data Fig. 3:**
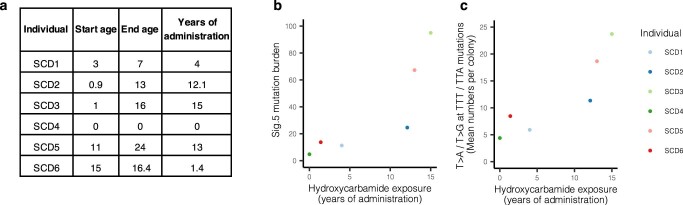
Hydroxycarbamide exposure. **a**, Approximate total length of hydroxycarbamide exposure in years for each SCD patient. **b**, Dot-plot showing the relationship between the mean mutation burden attributed to mutational signature ‘Sig.5’ per cell, and the years of hydroxycarbamide exposure in each individual. Of note, the extracted ‘Sig.5’ signature does not appear to be a ‘clean’ signature, demonstrating probable contamination by signature SBS19. The unique components of the ‘Sig.5’ signature are the T>A and T>G base pair changes seen at a TTT or TTA trinucleotide context. **c**, Here we consider *only* the T>A/ T>G mutations at a TTT or TTA trinucleotide context, the unique component of the extracted ‘Sig.5’ mutational signature. Dot-plot showing the relationship between the average number of such mutations per cell, and the years of hydroxycarbamide exposure in each individual. A regression analysis including age as a covariate suggests an additional 1.1 additional mutations per year of hydroxycarbamide exposure per HSC (95% CI, 0.3–1.9).

**Extended Data Fig. 4:**
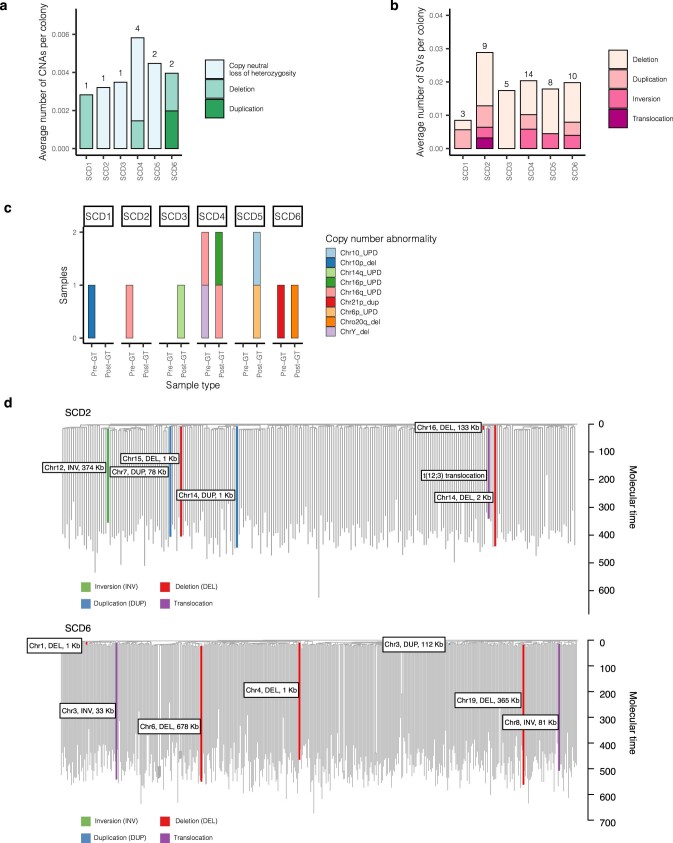
Copy number alterations and structural variants. Larger chromosomal changes were assessed, resulting in the identification of 42 structural variants (SVs) and 11 copy number abnormalities (CNAs) across all 2,592 pre- and post-GT colonies. **a**, Stacked bar plot showing the average number of CNAs per colony in each individual, divided by CNA type. Total numbers of CNAs in each individual is shown above the bar. Pre- and post-therapy samples are considered together. All alterations were acquired independently, as confirmed by the phylogeny. **b**, As per a, but for SVs. Here, two of the SVs in SCD2 were a single acquisition present in two colonies. **c**, Stacked bar plot showing specific CNAs and the samples they were found in, divided by individual and pre- / post-therapy. The bar fill represents the specific abnormality. A particular excess of SVs were seen in SCD2 who had 9/312 colonies (2.9%, 95% CI 1.3–5.4%) harboring an SV. **d**, The SNV-based phylogenies of SCD2 and SCD6, with the SVs overlaid on the branches during which they were acquired. Branches are colored by the type of SV. The 133Kb deletion in chromosome 16 in SCD2, and the 112Kb duplication in chromosome 3 in SCD6 can be timed to before 20 mutations of molecular time, equating to the first trimester of *in utero* development. del = deletion, dup = duplication, inv = inversion.

Somatic mutations accumulate in HSCs linearly over time, with approximately 14−18 SNVs and 0.65–0.77 indels acquired in each HSC per year^26–28^. In pre-GT samples, we observed a significant elevation in mutation burden in four of six patients compared to what would be expected for individuals matching these patients’ ages (Fig. 1a and Extended Data Fig. 2). Of note, the healthy control data used for comparison here are not ancestry-matched to our patient cohort, so we cannot exclude the possibility that other germline factors may influence mutation burden. Mutational signature analysis revealed evidence of the well-described ‘HSPC signature’ (ref. ^27^), but also several signatures not previously found in hematopoietic cells that accounted for the excess mutation burden in some individuals (Fig. 1b). There were no universal new mutational signatures present across all patients, indicating that the disease itself does not seem to be associated with one specific mutational process (Extended Data Fig. 2d). A new signature most notable for unusual T > A or T > G transversions in a TTA or TTG trinucleotide context (labeled ‘Sig.5’; Fig. 1b) was identified in a number of patients (Extended Data Fig. 3). Looking across patient history for a potential cause, the only parameter we found that was associated with this signature was hydroxycarbamide (HC) exposure (*P* = 0.02, linear regression including age as covariate), although a definitive relationship between mutational burden and HC was not established. Notably, absolute contributions of this signature to overall mutation burden are relatively small (Extended Data Fig. 2d). Other mutational patterns were observed in some patients (Extended Data Fig. 2), including the proliferation-associated signature SBS1 (patient SCD1) and SBS19 (unknown etiology, patients SCD2 and SCD3). Larger chromosomal abnormalities were also observed at slightly higher rates than expected for individuals of this age (Extended Data Fig. 4).

### Mutation burden and HSC relatedness before gene therapy

Patterns of shared and unique somatic mutations were next used to construct pre-GT phylogenetic trees for each individual (Fig. 1c). These phylogenetic trees provide data on the HSC lineage relationships between ancestors of the HSPCs sequenced. Branch points on these trees, termed ‘coalescences’, indicate historic stem cell self-renewal divisions where one HSC has given rise to two daughter HSCs. We were interested in establishing whether the trees of patients with SCD showed any evidence of postnatal expanded clones (operationally defined as an ancestral HSC from after in utero development that contributes >1% of colonies at the time of sampling^28^).

The pre-GT phylogenetic trees of all patients were highly polyclonal, similar to phylogenies from young healthy individuals, and in contrast to the patterns observed in elderly patients or those with a hematological malignancy^28,29^. Considering WGS data from 147–266 colonies per patient, we observed that almost all colonies were unrelated to one another following fetal development. We did not observe any clonal expansions, with no more than two colonies deriving from the same postnatal clone (<1% of the total number of colonies). These data suggest that steady-state hematopoiesis in younger patients with SCD is maintained by a large and diverse population of HSCs.

### Mutation burden in post-gene therapy HSCs

**Fig. 2:**
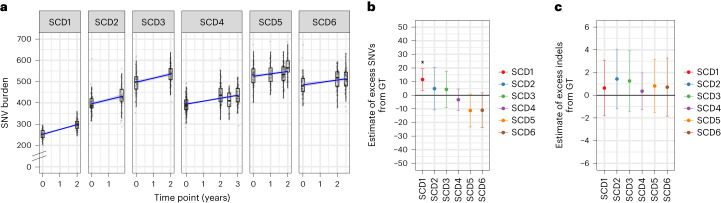
Gene therapy induces few additional somatic mutations. **a**, SNV mutation burdens of HSPC colonies (*n* = 1,564) from six individual patients plotted against the time point of colony sampling (relative to the GT procedure). The box-and-whisker plots show the distribution of mutational burden per colony per time point within each individual, with the boxes indicating median and interquartile range (IQR). The upper whisker extends from the hinge to the largest value no further than 1.5 × IQR from the hinge and the lower whisker extends from the hinge to the smallest value at most 1.5 × IQR of the hinge. The overlaid points are the jittered observed mutational burden of individual colonies. The solid blue line represents the inferred correlation between the mutation burden and the time point (simple univariate linear model), with the gray-shaded area showing the 95% confidence interval of this correlation. Time 0 represents data from samples taken at baseline for all patients. **b**, Estimate of the number of excess SNV mutations acquired from the GT procedure for individual patients. **c**, Excess indel mutations acquired from the GT procedure. For **b** and **c**, dots represent the difference in mean age-adjusted values between pre- and post-GT samples (*n* = 1,564 total colonies) and the bars show the 95% confidence interval of the estimated true difference between mean values (two-sided *t*-test). *P* values for SNV comparisons were 0.0051, 0.54, 0.52, 0.41, 0.067 and 0.090 for SCD1 to SCD6, respectively. *P* values for indel comparisons were 0.18, 0.19, 0.41, 0.61, 0.72 and 0.71 for SCD1 to SCD6, respectively. *P < 0.05.

**Extended Data Fig. 5:**
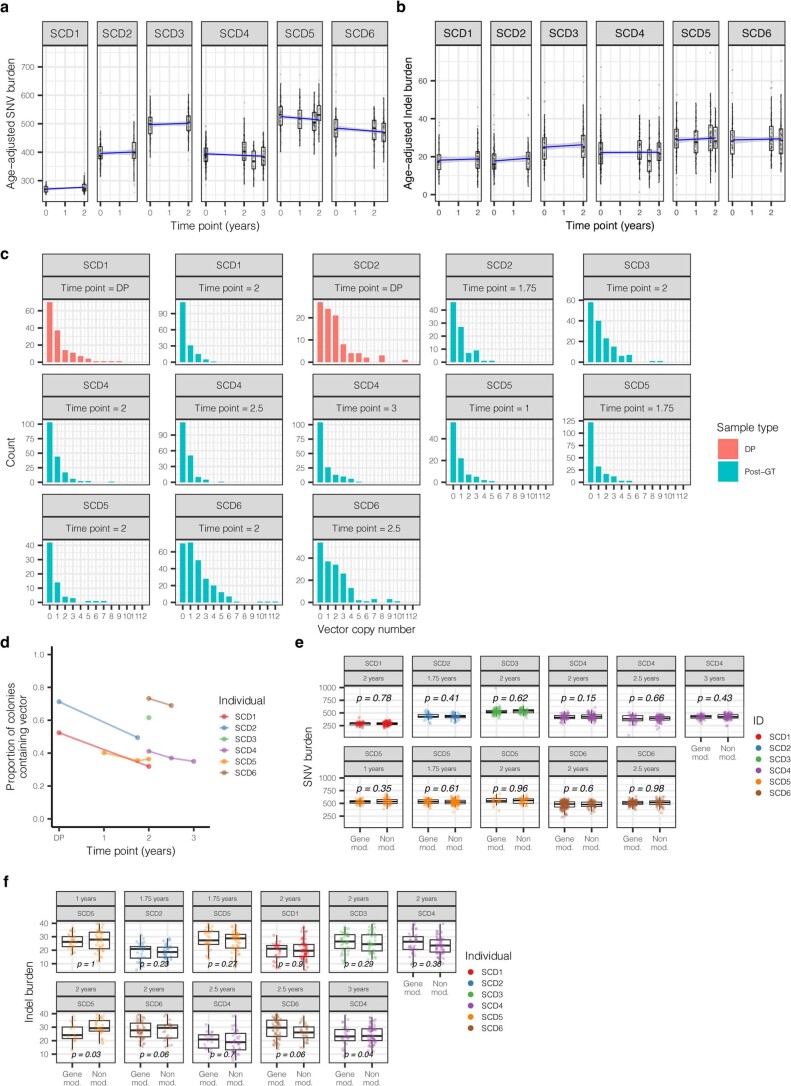
Mutation burdens and vector copy number of post-transduction samples. **a**, SNV mutation burdens of colonies from all patients (n=1,564 colonies total) plotted against the time point of colony sampling (relative to the gene therapy procedure), with post-therapy burdens corrected for the additional mutations expected from increased age, assuming 16.8 mutations per year per HSC. The box-and-whisker plots show the distribution of mutational burden per colony per time point within each individual, with the boxes indicating median and interquartile range. The upper whisker extends from the hinge to the largest value no further than 1.5 * IQR from the hinge and the lower whisker extends from the hinge to the smallest value at most 1.5 * IQR of the hinge. The overlaid points are the jittered observed mutational burden of individual colonies. The solid blue line represents the inferred correlation between the mutation burden and the time point (simple univariate linear model), with the gray shaded area showing the 95% confidence interval of this correlation. Time 0 represents data from samples taken at baseline for all patients. **b**, As per a, but of indel mutations. **c**, Barplot showing the number of post-therapy or donor product samples with different vector copy number values, split by individual and time point. **d**, Dot-plot showing the proportion of colonies that are transduced with at least one copy of the vector. This includes data from post-therapy and drug product colonies only. Where individuals have values from multiple time points, these are joined by a line to aid visualization. **e-f**, Box-and-whisker plots showing the corrected SNV and indel burdens for individual colonies from post-gene therapy time points (n=1,564 colonies total) separated by colonies with no evidence of vector integration (‘unmodified’), and those with at least one vector integration site (‘gene modified’). The boxes indicate median values and interquartile range. The upper whisker extends from the hinge to the largest value no further than 1.5 * IQR from the hinge and the lower whisker extends from the hinge to the smallest value at most 1.5 * IQR of the hinge. The overlaid points are the jittered observed mutational burden of individual colonies. The printed p-values relate to the significance of differences between the gene modified and non-modified colonies (two-sided t-test). DP = drug product.

Next, we compared HSC mutation burden pre- and post-GT to determine if the manipulations required for cell manufacturing, lentiviral integration and engraftment induce mutations. On average, mutation burdens from post-GT time points had increases of between 9 and 42 SNVs per HSC compared to pre-GT samples (Fig. 2a); however, when adjusted for normal aging, we observed no significant difference between pre- and post-GT time points for any patients except SCD1, who had an excess of 14 mutations above that expected for their age (7–21, 95% CI), equivalent to approximately 1 year of aging in an otherwise healthy individual (Fig. 2b and Extended Data Fig. 5a). There was no evidence of additional indels being induced by GT manipulations (Fig. 2c).

Alongside somatic mutation tracking, our approach allows concomitant identification of integrated vector sequences, thereby permitting us to distinguish gene-modified from unmodified HSPCs. For each colony, we determined whether the founder cell had been gene modified and quantified the number of vector copies integrated (Extended Data Fig. 5c). Overall, ~48% of colonies from post-GT samples were gene modified (range, 29–72%, 12–36 months post-transplantation). As reported in other GT trials, the proportion of modified HSPCs was higher in the drug product than in follow-up samples isolated from 12–36 months post-infusion (Extended Data Fig. 5d). Independent data from this clinical trial have shown that vector copy number has stabilized over the follow-up period for all patients^25,30^. No specific mutational signature was found in post-GT colonies and the mutation burden of gene-modified colonies was the same as that of unmodified colonies (Extended Data Fig. 5e,f). High doses of alkylating agents similar to busulfan have been shown to cause somatic mutations with specific mutational signatures^31,32^. Therefore, if any of the colonies in our dataset derived from non-transplanted clones that had survived the myeloablative busulfan conditioning^25^, we would expect to see evidence of this in their mutation profiles. The absence of such colonies suggests that the majority of post-GT colonies, including unmodified ones, were derived from transplanted clones. We cannot exclude the possibility that cells exposed to conditioning are less able to form colonies and are therefore under-represented in our dataset.

### HSC number and clonal relatedness post-gene therapy

**Fig. 3:**
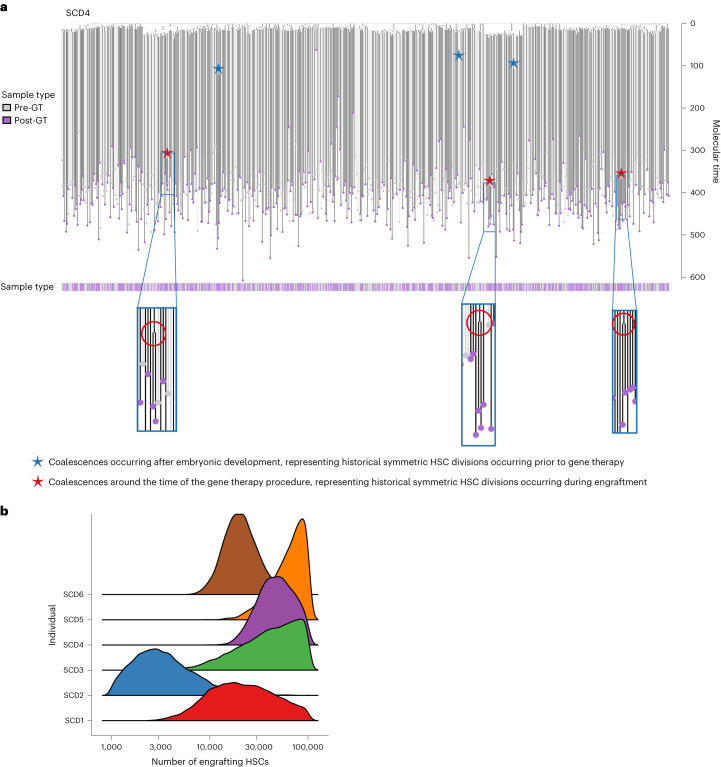
Combined phylogenies of pre- and post-gene therapy colonies and estimates of the number of engrafting long-term repopulating cells. **a**, Phylogeny of HSPC colonies sampled pre- and post-GT from the individual SCD4. Tips of pre-GT samples are shown in light gray, whereas those of post-GT samples are shown in purple. Branches from pre-GT samples only are shown in light gray and branches from post-GT samples (or both) are shown in dark gray. Branches are scaled according to the number of mutations allocated to that branch. Blue stars highlight post-embryonic late-branching events occurring before GT; red stars highlight post-embryonic branching events occurring after GT. **b**, Density plot showing estimates of the number of engrafting HSCs for each individual.

**Extended Data Fig. 6:**
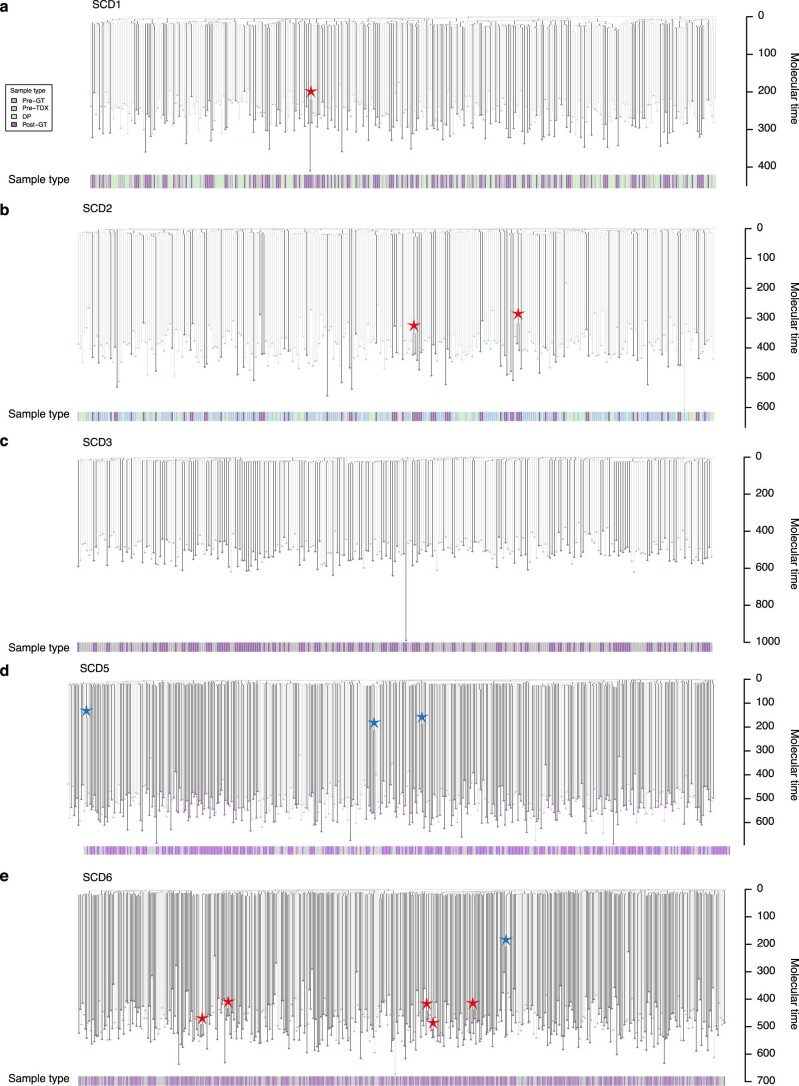
Post-gene therapy phylogenetic trees. **a-d**, Phylogeny of pre- and post- gene therapy colonies from SCD1 (**a**), SCD2 (**b**), SCD3 (**c**), SCD5 (**d**) and SCD6 (**e**). Tips of pre-therapy samples are light gray, while those of post-therapy samples are purple. Branches from pre-therapy samples only are colored light gray. Branches from post-therapy samples (or both) are in dark gray. Branches are scaled according to the number of SNVs allocated to that branch, termed ‘molecular time’. Blue stars highlight post-embryonic coalescences occurring prior to gene therapy. Red stars highlight post-embryonic coalescences occurring around the time of gene therapy. GT = Gene therapy. TDX = transduction. DP = Drug product.

**Extended Data Fig. 7:**
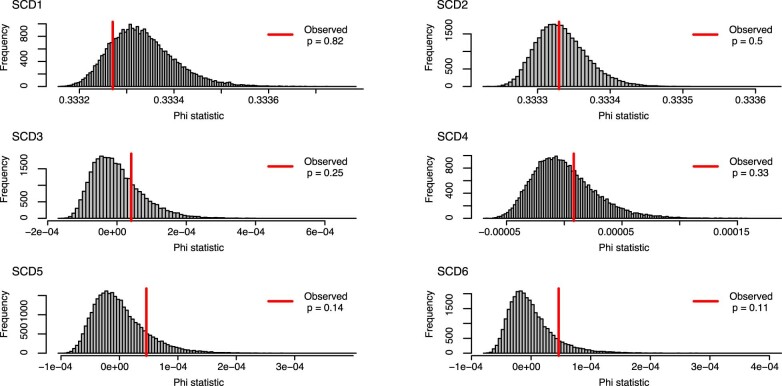
Analysis of molecular variance. Analysis of molecular variance (AMOVA) was used to test for clustering of post-therapy samples on the phylogenetic tree. Red lines show the observed phylogenetic clustering of pre- and post-therapy samples on the phylogenetic tree, as measured by the ‘Phi’ statistic. The significance of this statistic is obtained by comparing the value to the values obtained from random shuffles of sample labels (*n* = 1000) shown here as histograms. The one-sided p-values are the proportion of random shuffles with greater clustering than that observed in the data.

In addition to building phylogenetic trees for patients before GT, we explored HSC relatedness within post-GT samples. After constructing trees, we observed that post-GT HSPC samples mapped back across the entirety of the initial tree (Fig. 3a and Extended Data Fig. 6) with no significant phylogenetic clustering (Extended Data Fig. 7), indicating no selection for specific embryonic subsets of related HSCs.

**Extended Data Fig. 8:**
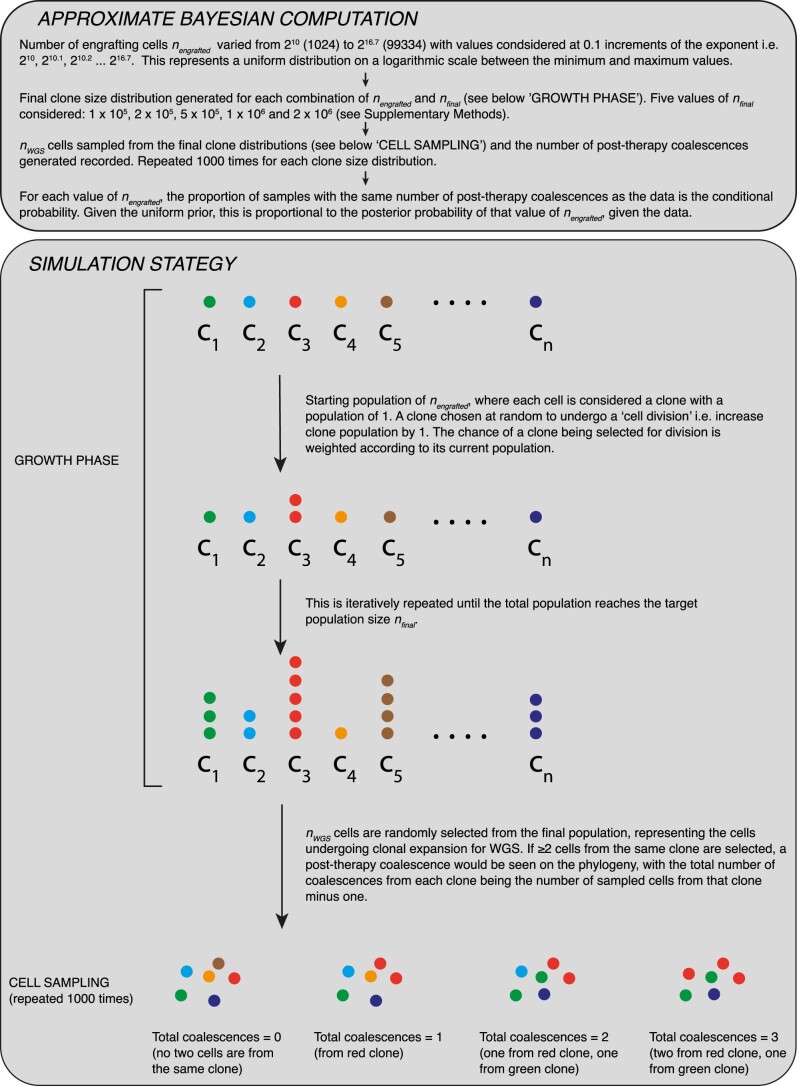
Approximate Bayesian Computation for inference of engrafting cell numbers. Schematic of the approximate Bayesian computation approach used for inferring the engrafting cell number for SCD3 and SCD4. This complements the text in Supplementary Methods.

**Extended Data Fig. 9:**
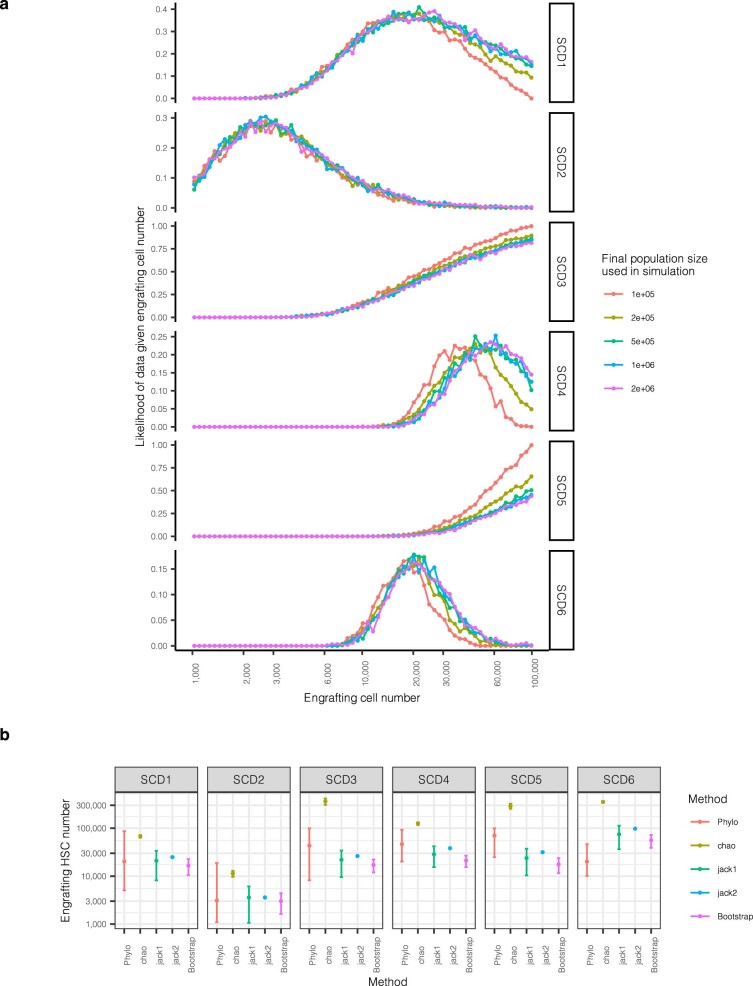
Analysis of molecular variance. **a**, Dot-plot showing the proportion of simulations where the number of post-therapy coalescences matches that observed in the data (as per Fig. 3c), split by the final HSPC population size used in the simulation. The values in Fig. 3c represent values averaged over the different population sizes. **b**, Estimates of engrafting HSC numbers using our method and 4 other methods using vector integration site diversity analysis. For our method (‘Phylo’), dots represent the median posterior value, and error bars the 95% posterior interval. For the vector integration site diversity methods, dots represent the point estimate, and error bars the approximate 95% confidence interval calculated as 1.96x the standard error on either side.

The number of engrafting HSCs in the GT procedure is not well established. Population bottlenecks, such as those occurring during transplantation of limited numbers of stem cells, leave characteristic features in the phylogenetic structure that can be used to estimate historic population sizes^24,33^. Accordingly, smaller numbers of transplanted stem cells would result in more late-branching events as this small population expands to repopulate the bone marrow. Illustrating this, Fig. 3a shows patient SCD4 with three post-GT late-branching events which can be used to estimate the transplanted HSC pool size (Fig. 3a and Extended Data Fig. 6; red stars). Using an approximate Bayesian computational (ABC) framework (Extended Data Fig. 8 and Online Methods), we estimated the number of engrafting long-term repopulating cells that remained active in the progenitor compartment at the time of sampling. We assume that engrafted clones that contribute new HSPCs 2–3 years post-transplantation have demonstrated long-term hematopoietic output as previously reported^34,35^ and can retrospectively be considered long-term repopulating cells. The estimates from the ABC revealed considerable variation between patients, with the lowest estimate for SCD2 of 3,100 (1,200–18,800, 95% prediction interval) and highest estimate for SCD5 of 70,240 (24,800–100,000, 95% prediction interval) (Fig. 3b and Extended Data Fig. 9a). Notably, SCD2 had the lowest infused CD34^+^ cell dose per kg (Table 1). Estimates were comparable to those obtained via standard vector integration site (VIS) analyses (Extended Data Fig. 9b).

### Driver mutations in pre- and post-gene therapy HSCs

**Fig. 4:**
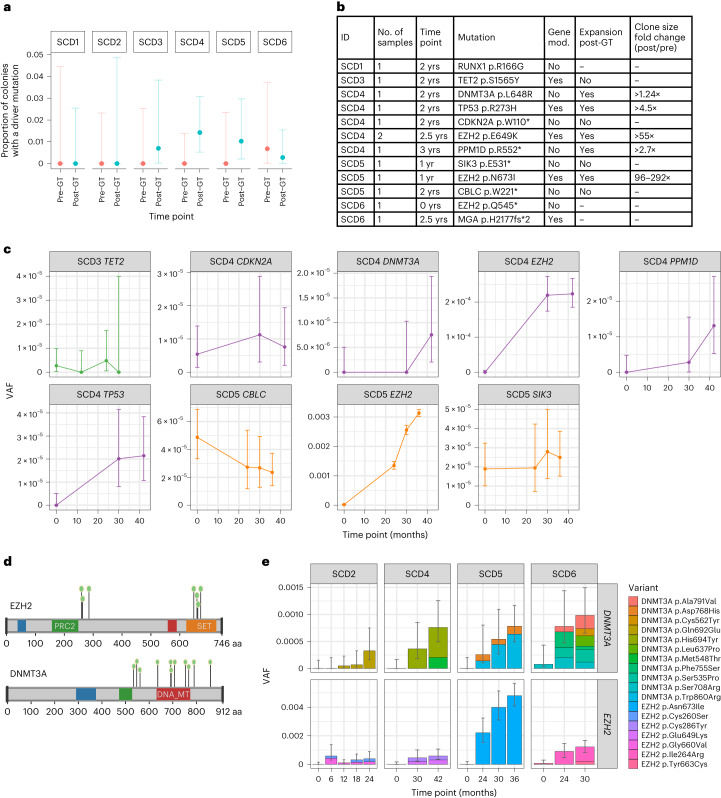
The proportion of colonies harboring driver mutations increases post-gene therapy. **a**, Dot-plot showing the proportion of HSPC colonies sampled pre- and post-GT with a potentially pathogenic driver mutation in each individual (*n* = 2,592 total colonies sampled). Pre-GT samples were taken at baseline for all patients; post-GT data include all post-GT time points analyzed for each patient. Dots show the exact proportion and error bars indicate the 95% confidence interval (exact binomial test). **b**, Table of potential driver mutations detected in the single-cell colony sequencing data. Where sequencing of pre- and post-GT samples was performed, we show whether the clone was substantially larger after GT and the fold change. The ‘time point’ column indicates when the samples were taken from each patient (years post-GT). The ‘gene mod.’ column indicates whether the mutation was found in a gene-modified or unmodified HSPC colony. **c**, Dot-plots showing the clonal trajectories of nine driver clones from pre-GT (time, 0, baseline only), through to the last available time of follow-up. The patient ID numbers and mutated gene are indicated for each plot. Dots show the exact VAF (number of variant reads divided by total coverage at that site) and error bars show the 95% confidence interval (binomial test). **d**, Lollipop plot showing the locations of altered amino acids in EZH2 (*n* = 7) and DNMT3A (*n* = 11) caused by missense mutations called in high-depth duplex targeted sequencing for individuals SCD2, SCD4, SCD5 and SCD6. **e**, Bar plots showing the total burden of *DNMT3A* (top) and *EZH2* (bottom) mutations from pre-GT (time point, 0) through to the last follow-up sample available. The center of the error bars is the sum of the VAFs of each individual mutation. Error bars show the 95% confidence interval of this value (Bayesian inference approach).

Recent occurrences of myeloid transformation events^20,21,36^ not associated with insertional oncogenesis have highlighted the need for more detailed information about genetic predisposition to leukemia and the potential occurrence of mutations in the post-GT pool of engrafting HSPCs. None of the patients in our study had detectable driver mutations in any follow-up samples using a Clinical Laboratory Improvement Amendments (CL1A)-certified 95-gene rapid heme panel with a variant allele fraction (VAF) sensitivity >1% (ref. ^37^). We surveyed individual colony genomes of all patients for the presence of potential cancer-associated mutations and identified 12 possible pathogenic mutations in *RUNX1*, *TP53*, *CDKN2A*, *DNMT3A*, *SIK3*, *EZH2* (three independent mutations), *TET2*, *CBLC*, *MGA* and *PPM1D* (Fig. 4a,b and Supplementary Table 2). All but one of these were detected in post-GT colonies and appeared in both modified and unmodified cells. Assessed together, these data revealed a post-GT increase in the proportion of colonies carrying a possible driver mutation from 1 in 1,161 (0.1%) pre-GT to 12 in 1,431 (0.8%) post-GT (*P* = 0.016; Fisher’s exact test). Although normal aging may contribute, the short follow-up periods of the post-GT samples (maximum 3 years) alone would not be anticipated to result in detectable increases in driver mutations.

**Extended Data Fig. 10:**
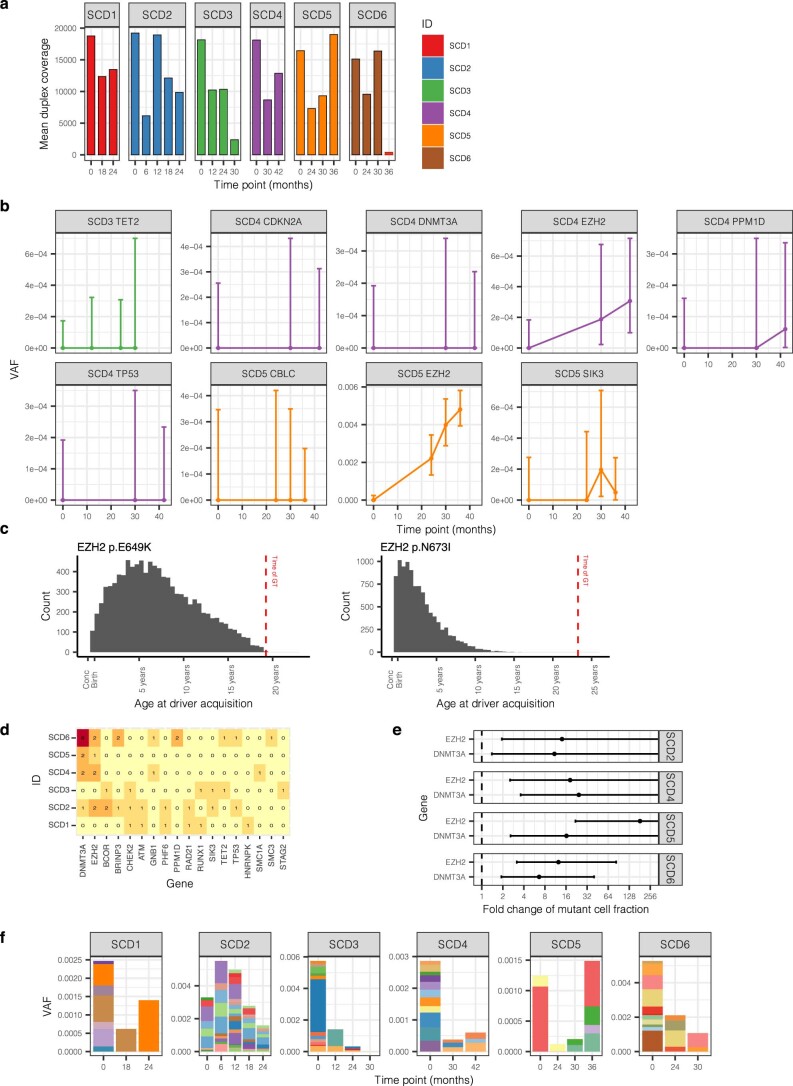
High depth targeted duplex sequencing. **a**, Mean duplex coverage across targeted sites by individual and time point. The 36 month sample from SCD6 failed. **b**, The driver mutation itself was directly detected for four mutations (PPM1D p.R552*, SIK3 p.E531*, EZH2 p.E649K and EZH2 p.N673I), and in all cases, this was in a post-GT sample. Here, we show the VAF of putative driver mutations through time, as in Fig. 4c, but for the driver mutation only, rather than the average across all mutations within the clone. Dots show the exact VAF (number of variant reads divided by total coverage at that site) and error bars show the 95% confidence interval (binomial test). **c**, The inferred time of acquisition of the two driver mutations with the highest clone VAFs (EZH2 p.N673I and EZH2 p.649K mutations), compared to the time of gene therapy, showing their likely acquisition prior to therapy. **d**, Heatmap of numbers of driver mutations per gene by individual. **e**, Dot-plot showing the estimated fold change of the fraction of cells harboring mutations in *DNMT3A* or *EZH2*. The dots show the median posterior values and the error bars the 95% posterior interval as estimated by Bayesian inference. Where error bars extend all the way to the right of the plot, there is no upper bound of the posterior interval. **f**, Burden of synonymous/ intronic mutations called in the duplex sequencing data, displaying very different trajectories to those of the putative *DNMT3A* and *EZH2* driver mutations shown in Fig. 4. GT = Gene therapy. VAF = Variant allele fraction.

To examine the acquisition of additional driver mutations in more detail, we performed targeted high-depth duplex sequencing^38,39^ on pre-GT and at least two post-GT bulk myeloid cell samples from each patient. As part of this panel, we targeted nine putative driver mutations identified in our individual colony analysis (Fig. 4b). For each branch with a putative driver, we also identified 40 additional unique SNVs to act as further indicators of that clone contributing to blood cell production (mean duplex depth of 12,392×; Extended Data Fig. 10a and Supplementary Table 3). The presence of the driver itself, or any of the additional 40 branch-specific mutations, allows us to identify how much that clone was contributing at the time of sampling. This method provides the power to detect clones with frequencies of <1 in 20,000 cells in most cases.

Using this approach, all nine driver-containing clones were detected in post-GT samples and five of nine driver-containing clones were detected in pre-GT samples (Fig. 4c and Extended Data Fig. 10b). The inability to detect some driver-containing clones in pre-GT samples is likely due to the level of detection of the assay rather than the non-existence of the clone. We used established methods to retrospectively determine the order of mutation acquisition within a clone. Given the depth of sequencing, this approach could be used for clones with a VAF > 0.05% (ref. ^40^). We could thus infer that the drivers from two of the four clones that were undetectable in the pre-GT samples were nonetheless likely to have been present before GT (Extended Data Fig. 10c). Following engraftment, the VAFs of five of nine driver-containing clones (PPM1D p.R552*, TP53 p.R273H, DNMT3A p.L648R, EZH2 p.E649K and EZH2 p.N673I) significantly increased by the final post-GT time point compared to pre-GT, with 55- and 95-fold minimum increases observed for the two *EZH2* mutant clones (Fig. 4b). More modest increases of 1.24-, 2.7- and 4.5-fold were seen for the *DNMT3A*, *PPM1D* and *TP53* mutant clones, respectively. Of note, the EZH2 p.N673I clone in SCD5 demonstrated ongoing expansion up to the final 3.5-year post-GT time point (Fig. 4b,c). Notably, for all of these mutations, the increases are below the sensitivity threshold of the clinical targeted sequencing panel used during follow-up and the clinical relevance is not known at this point.

In addition to tracking mutations previously identified in the tree-building phase of this study, we also performed de novo mutation calling from the duplex sequencing data across a panel of 39 myeloid cancer-associated genes (Supplementary Table 4). This identified 49 somatic mutations predicted to have at least a moderate functional impact (Extended Data Fig. 10d and Supplementary Table 5). *DNMT3A* (*n* = 11) and *EZH2* (*n* = 7) were most commonly mutated, with mutations in the latter clustered in two specific gene regions (Fig. 4d). The burden of *DNMT3A* or *EZH2* mutant cells showed a significant increase through gene therapy in four of six individuals, from undetectable pre-GT, up to ~0.1% combined VAF post-GT (equivalent to ~ 1 in 500 cells) corresponding to a 6- to 180-fold increase (Fig. 4e and Extended Data Fig. 10e). Two *EZH2* mutations had the largest post-GT VAFs (EZH2 p.E649K, 0.03% (95% CI 0.01–0.07%) at 3.5 years in SCD4, EZH2 p.N673I, 0.5% (95% CI 0.4–0.6%) at 3 years in SCD5). This trend was not seen in synonymous or intronic mutations (Extended Data Fig. 10f), suggesting that these increases are the result of positive selection. Combined, these data suggest that the ex vivo manipulations during the GT procedure or the process of engraftment selects for clones with pre-existing driver mutations.

## Discussion

Our large-scale whole-genome study of >2,500 single-cell-derived colonies has revealed a number of genomic features in the context of SCD, several of which have wider implications for the HSC GT field. First, some individuals with SCD have additional genomic damage at baseline. Second, we estimate that up to tens of thousands of HSCs contribute to both pre- and post-GT hematopoiesis and clonal expansions larger than 1% are not observed in these patients. Third, somatic mutation burden does not seem to be substantially increased as a result of the GT procedure. On the other hand, increased frequencies of clones harboring driver mutations post-GT suggest selective pressure on HSPC clones with increased fitness, rather than increased gene therapy-related mutation acquisition, as a potentially important mechanism for clonal expansion. This latter point indicates a need to understand the various aspects of the GT process including mobilization, ex vivo manipulation, transplantation and engraftment-based expansion, which may impose a selective pressure on different HSC clones and several of these processes are common to different types of GT approaches (viral vector-based and gene-based editing strategies). Although the relevance of clonal expansion in the setting of GT to the risk of hematological malignancy is currently unknown, our data reinforce the need for long-term follow-up for any patient receiving GT.

Previous work has suggested that individuals with SCD may be at increased risk of developing myeloid malignancies^41,42^. While we detected very few myeloid neoplasm-associated mutations at baseline in HSPCs, we did observe an increased total number of mutations per HSPC in four of six patients compared to healthy cohorts. Consistent with other recent reports^43,44^, the specific mutagenic processes driving this seem to be heterogeneous between patients, with no unique molecular signature associated with SCD. Elevated HSPC proliferation due to high red-cell turnover and common treatments may contribute for some individuals, though further study is needed to investigate these relationships.

Our approach of WGS and phylogenetic reconstruction of single-cell-derived colonies is particularly powerful for studying post-GT samples as it permits the identification of driver mutations in both gene-modified and unmodified progeny, the latter of which researchers are typically blind to unless the clone has expanded substantially. We detected driver mutations equally in clones with or without vector integration, suggesting that viral integration is not the primary cause of the increased frequency of driver mutations we observed post-GT in some patients. We also found that the GT procedure itself does not contribute large numbers of additional somatic SNV mutations. This may seem surprising given the stress of expansion required for hematopoietic reconstitution, but it accords with data from allogeneic hematopoietic cell transplants (HCTs) where no additional HCT-associated mutations were observed^45,46^. It is further consistent with our finding of large numbers of engrafting cells, which might result in few additional divisions per cell, combined with a low rate of cell division-associated mutations in HSCs^47^. Nonetheless, these data demonstrate the importance of monitoring for clonal expansions in both gene-modified and unmodified clones, as previous experience has shown in at least one case that malignancy can develop from unmodified clones. This may also be relevant to other transplantation settings as blood cancers have also been reported in patients with SCD treated with HCT^48–54^.

While GT did not cause substantial numbers of additional mutations, our de novo mutation tracking data indicate that the GT procedure promotes the growth of pre-existing driver mutations, leading to a selection of clones that increased in size from extremely small (approximately 1 in 30,000 cells) to slightly larger (up to 1 in 100–200 cells). While the fraction of cells with driver mutations remains small, this represents a >100-fold expansion in a period of ~3 years. While it is formally possible that surviving clones may have expanded neutrally after the population bottleneck induced by the GT process, it is unlikely that this can fully explain these expansions, as we do not see the same trajectories for non-synonymous and intronic mutations called using the same strategy and some of the expansions observed are highly atypical of neutral expansions (>100-fold) given that the estimated bottleneck is >10% (roughly 10,000–50,000 cells from the estimated 100,000–200,000 active HSCs in other studies^24,55,56^). As a comparison, this is considerably faster than expanding clones found in patients with myeloproliferative neoplasms, for which doubling times in this setting of malignancy are estimated at 8 months (equivalent to a ~22-fold expansion in 3 years)^29^. In the context of GT, this rapid expansion rate may be a transient consequence of marrow repopulation, but even so, it increases the pool of cells with potential to undergo further clonal evolution. In our patient group, *DNMT3A* and *EZH2* mutations demonstrated the largest clonal expansions. Mutations in these genes have been associated with clonal hematopoiesis and myeloid disorders^57–61^, though neither were reported to be mutated in patients who experienced post-GT myeloid malignancies^20,21^, emphasizing the lack of understanding of the clinical relevance and predictive nature for these particular clones with VAFs <1%. Nevertheless, it suggests that some aspect of the GT process, even in the absence of vector integration, may exert selective pressure on particular clones with greater fitness, leading to clonal expansion. This hypothesis is further supported by the observation that mutations in *EZH2* and *DNMT3A* were not enriched in a similar phylogeny-building study looking at mutations in patients who had undergone allogeneic HCT^62^. Notably, *EZH2* mutations seem to be under clear positive selection in our dataset, but are rare in age-related clonal hematopoiesis. This highlights that mutations selected for in the setting of GT are not restricted to those associated with clonal hematopoiesis. The relevance of this phenomenon to myeloid malignancy following GT needs further study, including long-term follow-up.

Overall, our findings highlight an elevated mutation rate in some patients with SCD and positive selective pressure on HSCs containing pre-existing driver mutations as mechanisms that could increase leukemia risk in GT trials for SCD. This has a range of clinical implications. First, it raises the question of whether GT candidates should be screened for driver mutations. Our data suggest that pre-existing clones are often well below the detection limit of standard clinical sequencing technologies, making screening by these methods limited in utility. Equally, however, there is no firm evidence linking low-VAF mutations (those detectable only by highly sensitive sequencing platforms) with increased cancer risk. Discussion is therefore needed to determine whether high-sensitivity methods should be used to screen patients and limit eligibility for potentially curative autologous therapies. Second, our study highlights the importance of minimizing the risk of acquiring driver mutations before GT. To this end, GT may be considered in younger age groups, although this needs to be weighed against the potential risks of early busulfan exposure given the greater remaining lifespan. Finally, the development of a better understanding of the specific processes contributing to the selective expansion of clones harboring driver mutations, with the intent of minimizing these processes, would greatly benefit the field.

## Methods

### Patient samples and in vitro expansion of single HSPCs

Peripheral blood (PB) and/or bone marrow (BM) mononuclear cells (MNCs) were obtained from six consented patients with clinically severe SCD currently enrolled in clinical trial NCT03282656 (Boston Children’s Hospital; https://clinicaltrials.gov/ct2/show/NCT03282656). Briefly, patients were treated with 240 μg kg^−1^ of plerixafor and CD34^+^ cells were collected for drug product manufacturing^25^. After transduction and testing of cells with the BHC-BB694 BCL11A shmirR lentiviral vector, trial participants received fully myeloablative intravenous treatment of busulfan for four consecutive days before transduced CD34^+^ cells were infused^25^. Patient samples were selected for this study based on (1) the availability of a large number of pre-GT colony samples ready for sequencing and (2) the availability of >1-year post-transplantation samples. Fresh or frozen pre-GT BM, mobilized PB or pre-transplantation transduced CD34^+^ cells and post-transplantation follow-up BM and PB samples, where available, were thawed and plated as a single-cell suspension (500 cells per well for CD34^+^ BM and CD34^+^ mobilized PB; 750,000 cells per well for PB-MNCs) into MethoCult H4434 (cat. no. 04434, STEMCELL Technologies). Resulting single progenitor-derived colonies were picked at 14–21 d into either Dulbecco’s phosphate-buffered saline (cat. no. D8537, Sigma Aldrich) or Proteinase K buffer (cat. no. KIT0103, Arcturus PicoPure DNA extraction kit, Applied Biosystems). DNA was extracted using the Arcturus PicoPure DNA extraction kit and stored at −20 °C for downstream WGS. While biases may exist in terms of which HSPCs give rise to colonies in this assay, the expansion of HSPC-derived cells was required to provide enough genetic material for WGS.

### WGS and identification of somatic mutations

Library preparation and WGS was performed using a method developed for low quantities of input DNA, as previously described^63^. Paired-end sequencing reads (150 bp) were generated using the Illumina NovaSeq 6000 platform to a target coverage of 10–15×, with a subset of samples sequenced to a higher target coverage of 30–40×. SNVs and indels were called against an unmatched synthetic reference genome using standard pipelines^64,65^. BWA-MEM was used to align sequences to the human reference genome (v.0.7.17, NCBI build 37). Following alignment, SNVs and indels were called against an unmatched synthetic reference genome using the Sanger in-house pipelines CaVEMan (v.1.13.14) and Pindel (v.3.3.0), respectively, using standard settings^64,65^. A total of 2,030 colonies underwent WGS. Of these, 10 were excluded due to low sequencing coverage (<4×), 291 were excluded as being non-clonal and 149 were excluded as being duplicates from the same colony, leaving a total of 1,580 included in the final analysis (Online Methods and Extended Data Fig. 1b).

For all mutations passing quality control filters in CaVEMan and Pindel, matrices of variant and normal reads were determined for all HSPC colonies using the cgpVAF software (v.5.6.1; https://github.com/cancerit/vafCorrect). Post hoc filtering steps were then applied to (1) remove artifacts associated with the low-input library prep pipeline such as cruciform DNA structures (SangerLCMFiltering, v.1.03; https://github.com/MathijsSanders/SangerLCMFiltering); (2) remove germline SNVs using an exact binomial filter to aggregate counts of normal and variant reads across all samples^66^; (3) remove low-frequency artifactual mutations for which count distributions across samples did not come from an over-dispersed β-binomial distribution^67,68^; (4) remove mutations at sites with abnormally high or low mean coverage (mean depth below 8× or over 40×); (5) remove mutations inconsistent with a true somatic mutation as determined by aggregating normal and variant reads from positive samples (≥3 variant reads) and then using a one-sided exact binomial test to filter those with a *P* value < 0.001; and (6) retain mutations if at least one sample met minimum thresholds for variant read count and total depth and had a VAF > 0.2. Additionally, the data for some colonies were removed from the dataset due to low sequence coverage (coverage <4×, 10 samples), the presence of technical duplicates (149 samples) and evidence of non-clonality or contamination (291 samples). A peak VAF threshold of <0.4 was used to identify data from mixed colonies, with additional samples removed following phylogeny-building by checking mutation VAFs against the phylogeny and removing those inconsistent with a clonal sample. Custom R scripts used for these filtering steps are available (https://github.com/mspencerchapman/Gene_therapy). The following open source R packages were used in the analyses presented throughout this paper: data.table (v.1.12.8), ggplot2 (v.3.3.0), stringr (v.1.4.0), seqinr (v.3.6-1), tidyr (v.1.0.2), dplyr (v.0.8.5), plotrix (v.3.7-7), phangorn (v.2.5.5), RColorBrewer (v.1.1-2), ape (v.5.3), phytools (v.0.6–99), VGAM (v.1.1-2), gridExtra (v.2.3) and pheatmap (v.1.0.12).

### Identification of non-clonal samples

Hematopoietic colonies embedded within methylcellulose may grow into one another or derive from more than one founder cell, resulting in colonies that are not single-cell-derived. As these samples interfere with phylogeny building and have lower numbers of called mutations, they were excluded from downstream analysis. Detection of such colonies was conducted in two steps. The first step was based on the principle that somatic mutations from clonal samples should have a peak VAF density of 0.5. Therefore, following exclusion of germline mutations and recurrent artifacts using the exact binomial and β-binomial-filtering steps, the VAF distribution of positive mutations in a sample were assessed. Samples with a maximum VAF distribution density <0.4 (corresponding to a sample purity of <80%) were excluded. The second step was performed following a first iteration of phylogeny building using all samples passing the first step. Each sample was tested against the phylogeny to see whether the mutation VAFs across the tree were as expected for a clonal sample. A clonal sample should have either branches that are ‘positive’ (mutation VAFs ~0.5) or ‘negative’ (mutations VAFs ~0). Therefore, for each branch in each sample, variant and total read counts were combined across all branch mutations. These counts were then tested for how likely they were to come from either (1) at least that expected for a heterozygous somatic mutation distribution, with some contamination allowed (one-sided exact binomial test, alternative hypothesis = less than probability, probability = 0.425); or (2) no more than that expected for absent mutations, with some false positives allowed (one-sided exact binomial test, alternative hypothesis = greater than probability, probability = 0.05). If samples had any branches with read counts that were highly inconsistent with both tests (maximum *q* value < 0.05, Bonferroni correction) or had three or more branches that were minorly inconsistent with both tests (maximum *P* value 0.05, no multiple hypothesis testing correction) the sample was considered non-clonal and excluded. A second iteration of phylogeny building was then performed without the non-clonal samples. As indicated, these steps have a degree of tolerance of minimally contaminated samples and samples with >80–85% purity will generally be retained; however, even this lower level of contamination will have an impact on the sensitivity of mutation calling and therefore sample purity was taken into account for mutation burden correction (see below).

### Identification of colony duplicates

Some hematopoietic colonies grown in methylcellulose have an irregular branching appearance and are easily misinterpreted as multiple separate colonies. This may result in several samples being inadvertently picked from the same colony. Such samples seem highly related on the phylogenetic tree, with only a few private mutations, representing predominantly in vitro-acquired mutations. Recognition of these duplicates is aided by the fact that (1) in many cases, duplicates are picked into adjacent/nearby wells, as colony picking is performed systematically around the well, and (2) in most biological scenarios, such highly related sample pairs are extremely rare due to the larger short-term HSC/HSPC pool^28^; however, the first point may not always be true and in the setting of a recent transplantation procedure we expect there to be more genuine closely related samples representing HSC/HSPCs that have undergone symmetric cell divisions during BM repopulation. Given that the number of post-therapy coalescences is crucial in estimating the number of engrafting stem cells, accurate identification of colony duplicates was essential.

We therefore employed a strategy based on assessing the mutational signatures of private mutations (Supplementary Fig. 1). For colony duplicates, private mutations represent in vitro-acquired mutations, whereas for sample pairs with close in vivo relationships, they represent in vivo-acquired mutations. These have distinct mutational signatures. We first defined the in vitro signature using mutations from confident duplicate pairs that are those from adjacent/nearby wells. We then used the function ‘fit_to_signatures’ from the R package MutationalPatterns (v.3.14; https://doi.org/doi:10.18129/B9.bioc.MutationalPatterns) on each set of private mutations, using only the in vitro signature and ‘BM signature’ to define optimal contributions of these two signatures that best fitted the data (Extended Data Fig. 8a,b). Sample pairs where either sample had <15 mutations contributed by the BM signature were defined as colony duplicates.

### Phylogenetic tree construction and branch assignment

Phylogenetic trees were reconstructed as previously described^55^.

### Mutation burden correction

We used two different approaches to correct for sequencing coverage and colony purity. The ‘asymptotic regression’ correction method and the ‘sensitivity for germline polymorphisms’ correction method. Both use the ‘peak VAF’ measure, either to exclude lower purity samples from the analysis or to incorporate into the correction itself. This is defined here as the VAF value with the maximum density, assessing across all somatic mutations called in that sample and is a good measure of purity in higher coverage samples.

(i) Asymptotic regression
  We used this method for comparisons with published datasets of non-diseased individuals, which used the same method^55,69^. For clonal samples, the number of called mutations increases with coverage initially, but then plateaus once the coverage reaches levels of ~30×, at which point the majority of mutations within callable regions of the genome are detected. For each individual we selected ten pre-GT samples to be sequenced to a higher 30–40× WGS coverage. We similarly performed higher coverage WGS for ten post-GT samples for SCD3 and SCD4 for one post-therapy time point (2 years and 1 year, respectively). Using the ‘NLSstAsymptotic’ function from the R stats package, we fitted an asymptotic regression model to the relationship between numbers of called mutations and sequencing coverage, which we then used to correct the mutation burden for samples from the same individual/time point up to the level expected for 30× of sequencing coverage. Given that such a correction does not take into account differences in sample purity, we only included those samples with evidence of high purity (peak VAF > 0.46) and coverage (≥10×) in this correction step.
(ii) Sensitivity for germline polymorphisms
  This method was used to estimate the number of gene therapy-induced mutations and to correct phylogeny branch lengths. It uses the sensitivity for calling germline single-nucleotide polymorphisms (SNPs) or indels as a surrogate for the sensitivity for calling somatic mutations and thereby correct for sequencing coverage. This approach has the advantage of being applicable to all samples even in the absence of having a reference set of higher coverage samples and can be applied to the phylogeny to correct branch lengths. We also incorporated a sample purity correction step.

For each individual, reference sets of germline polymorphisms (separate sets for SNVs and indels) were defined. These were mutations that had been called in many samples (as mutation calling was performed against an unmatched synthetic normal) and for which aggregated variant/reference mutation counts across samples from an individual were consistent with being present in the germline. These were identified using the same exact binomial test as was used for filtering germline variants from the somatic mutation identification pipeline. In all cases the number of germline SNPs in the set was >100,000. For each sample, the proportion of germline SNPs that were called by CaVEMan and the LCM filtering pipelines was considered the ‘germline SNV sensitivity’ and the proportion of germline indels that were called by Pindel was the ‘germline indel sensitivity’. For pure clonal samples, the sensitivity for germline variants should be the same as for somatic variants. Therefore, for samples with a peak VAF > 0.48 (corresponding to a purity of >96%), this germline sensitivity was also considered the ‘somatic variant sensitivity’ and was used to correct the number of somatic variants; however, for less-pure samples (purity 80–96%), the sensitivity for somatic variants will be lower than for germline variants as they will not be present in all cells of the colony. Therefore, an additional ‘clonality correction’ step was applied. The expected number of variant reads sequenced for a heterozygous somatic mutation in a non-clonal sample will be *n*_*v*_~Binomial(*N,p*) where *N* is the sequencing coverage at the mutation position and *p* is the sample peak VAF (rather than *p* = 0.5 as is the case for a pure clonal sample). The likelihood of the mutation being called given *n*_*v*_ variant reads and *N* total reads was taken from a reference sensitivity matrix. This matrix was defined from the germline polymorphism sensitivity data across 20 samples, where for all combinations of *n*_*v*_ and *N*, the proportion of mutations called in each sample’s final mutation set was assessed. The sequencing coverage distribution across putative somatic mutations was considered the same as that across the germline polymorphism set. Therefore, for each value of *N* (the depths across all germline polymorphisms in that sample), a simulated number of variant reads *n*_*v*_ was taken as a random binomial draw as described above, and whether this resulted in a successful mutation call taken as a random draw based on the probability defined in the sensitivity matrix. The total proportion of simulated somatic mutations successfully called was defined as the ‘somatic variant sensitivity’ for that sample.

The somatic variant sensitivities were then used to correct branch lengths of the phylogeny in the following manager. For private branches, the SNV component of branch lengths was scaled according to:

$${n}_{\mathrm{cSNV}}=\frac{{n}_{\mathrm{SNV}}}{{p}_{i}}$$

Where *n*_cSNV_ is the corrected number of SNVs in sample *i*, *n*_SNV_ is the uncorrected number of SNVs called in sample *i* and *p*_*i*_ is the somatic variant sensitivity in sample *i*.

For shared branches, it was assumed (1) that the regions of low sensitivity were independent between samples and (2) if a somatic mutation was called in at least one sample within the clade, it would be ‘rescued’ for other samples in the clade and correctly placed. Shared branches were therefore scaled according to:

$${n}_{\mathrm{cSNV}}=\frac{{n}_{\mathrm{SNV}}}{1-\prod _{i}(1-{p}_{i})}$$

Where the product is taken for 1 − *p*_*i*_ for each sample *i* within the clade. Neither of these assumptions are entirely true. First, areas of low coverage are non-random, and some genomic regions are likely to have below average coverage in multiple samples. Second, while many mutations will indeed be ‘rescued’ in subsequent samples once they have been called in a first sample, because the treemut algorithm v.1.1 for mutation assignment goes back to the original read counts and therefore even a single-variant read in a subsequent sample is likely to lead to the mutation being assigned correctly to a shared branch, this will not always be the case. Sometimes samples with very low depth at a given site will have 0 variant reads by chance. In such cases, a mutation may be incorrectly placed. These factors both mean that the approach may under-correct shared branches, but it is a reasonable approximation. SNV burdens corrected by this approach were then taken as the sum of corrected ancestral branch lengths for each sample, going back to the root.

### Mutational signature extraction

Mutational signatures present in the data were identified by performing signature extraction using a hierarchical Dirichlet process as implemented in R package HDP (v.0.1.5; https://github.com/nicolaroberts/hdp). This produced six signatures, labeled Sig. 1–6 (Fig. 1 and Extended Data Fig. 2). Mutational signatures that were similar to known signatures or appeared as composites of known signatures were re-labeled accordingly. Only Sig. N5 had no resemblance to any known signatures and was therefore classed as ‘new’. All mutational signatures reflect underlying mutational processes that have been active in the HSPC colonies and contributed to the somatic mutation burden. Each branch on the phylogeny was treated as an independent sample and counts of mutations at each trinucleotide context were calculated. Branches with <50 mutations were excluded, as below this threshold random sampling noise in the mutation proportions becomes problematic.

Plots of signature contributions in each sample in Fig. 1c represent the weighted means of signature contributions of individual branches included within the sample (weighted by the branch length), with final values then scaled by the sample total mutation burden to reflect the absolute signature contributions. Notably, branches of <50 mutations, primarily early embryonic branches and private branches of duplicate colonies, were not included in this assessment of sample signatures as they had been excluded from the signature extraction step. This means that processes primarily operative in embryogenesis are under-represented in these estimates.

### Correction for in vitro-acquired mutations

In general, in vitro-acquired mutations acquired after the first 1–2 cells divisions of colony growth will be present in <1 in 4 cells within the colony, with expected VAFs of <0.125. The vast majority will therefore be excluded from the final somatic mutation sets by including a VAF cutoff of >0.2. This means that few in vitro-acquired mutations are expected within the final mutation set. Indeed, studies in fetal samples with very low mutation rates have estimated the number of in vitro mutations passing similar filtering steps to be ~four per colony^67^, and other studies have not attempted to correct for in vitro mutations, including the reference data from healthy individuals used as comparison^24,28^. Nevertheless, we wanted to make sure that the excess somatic mutation burden observed in our cohort was not related to increased rates of in vitro mutations. Therefore, we first defined an expanded set of nine reference mutational signatures. This included the seven mutational signatures extracted by HDP: the five putative in vivo signatures (Fig. 1b) and two putative in vitro signatures (Extended Data Fig. 2a); an ‘embryonic signature’ resembling SBS1, which was defined by combining the mutations from embryonic branches across individuals (those in which the entire branch is <50 mutations of molecular time); and an ‘in vitro signature’ defined by combining the mutations across the private branches of colony duplicates across individuals (Extended Data Fig. 8a). We then refitted the complete set of mutations within each sample to the optimal linear combination of these reference signatures using the function ‘fit_to_signatures’ from the R package MutationalPatterns (v.3.14, 10.18129/B9.bioc.MutationalPatterns). Contributions from any of the putative in vitro signatures (N0, N6 or ‘in vitro signature’) were then subtracted from the mutation burdens of each sample.

### Lineage mixed-effects model to assess increase in mutation rate from SCD

To formally assess the degree to which SCD increases the mutation acquisition rate we used a linear mixed-effects (LME) regression approach. We created a combined dataset of colony mutation burdens, ages and disease status from our pre-GT data and a reference dataset of hematopoietically healthy individuals^28^. This study, which we used as a reference dataset in several analyses, looks at a cohort of healthy adults, from whom sequencing data were derived from colonies that were grown from sorted CD34^+^CD38^−^ HSCs/multipotent progenitors (MPPs). Using the lme function from *R* package ‘nlme’ (v.3.1; https://cran.r-project.org/package=nlme) we first fitted a LME using only age as a fixed effect and individual as a random effect. We then fitted a second LME model adding in an age–disease status interaction term to assess whether this significantly improved the model and the magnitude of the excess mutation rate accounted for by having SCD. The addition of the interaction term did not significantly improve the model for SNV mutations or indels.

### Assessing vector copy number and vector integration sites

A custom human reference genome was defined by adding the anticipated vector integration sequence to the GRCh37 reference genome as an additional contig. All sample bam files were then remapped against this new reference using bwa-mem2 (v.0.7.17; https://github.com/bwa-mem2/bwa-mem2). Vector copy number was determined from the mean coverage across the vector sequence, which was determined using SAMtools and then normalized by the coverage in the rest of the genome (the vector coverage was divided by 0.5 × average autosomal coverage). This was further corrected for mismapping to the vector integration sequence by subtracting the average vector copy number from pre-transduction samples (this was approximately 0.3). Reassuringly, this yielded values that clustered around integers (Extended Data Fig. 1a).

To determine the approximate VIS, we first created a subsetted bam file for each sample. This contained only reads mapping to the standard reference genome, but whose pairs mapped to the vector integration sequence. Sites of recurrent mismapping across samples were filtered. Reads mapping to the same chromosome were clustered by position using the function ‘Ckmedian.1d.dp’ from R package ‘Ckmeans.1d.dp’ v.4.3.4, with potential *k* values ranging from 1 to 3. Clusters with close by positions (central positions <1 kb apart) were merged. Any cluster with at least four assigned reads was considered a VIS. In general, vector copy number and detected numbers of vector integration sites had high correlation.

### Inference of engrafting cell numbers

We inferred plausible numbers of engrafting cells for all patients using an approximate Bayesian computation (Extended Data Fig. 8). First, clone size distributions were simulated in ‘R’ from varying numbers of engrafting cells (*n*_engrafted_, where each engrafting cell is considered a ‘clone’) assuming growth via a birth process^70^. We defined a starting vector of length *n*_engrafted_ with all elements equal to 1 representing the initial sizes of engrafting ‘clones’. Clones were then grown by iteratively incrementing a randomly selected clone by 1, with the probability of a clone being selected proportional to its population after the previous increment. This was continued until a final population *n*_final_ was reached. Possible engrafting cell numbers *n*_engrafted_ were considered between 210 (1,024) up to 2^16.6^ (99,334), effectively giving a uniform prior between these values. For each value of *n*_engrafted_, a starting phylogeny of *n*_engrafted_ cells was taken as the starting tree, which was then grown up to a final population of active HSPCs *n*_final._ The size of each clone was then extracted from the phylogeny.

For each combination of *n*_engrafted_ and *n*_final_, samples of ‘cells’ were randomly drawn from this final population (*n* = 1,000) and the number of anticipated post-therapy coalescences inferred from the number of times that the same clone was sampled more than once. The number of sampled cells matched the number of post-therapy colonies undergoing WGS for each individual (143 for SCD1, 74 for SCD2, 143 for SCD3, 420 for SCD4, 292 for SCD5 and 358 for SCD6). Random draws from distributions with the same *n*_engrafted_ were pooled and the proportion of random draws with the same number of post-therapy coalescences as the data (1 for SCD1, 2 for SCD2, 0 for SCD3, 3 for SCD4, 0 for SCD5 and 5 for SCD6) was taken as the likelihood of that value of *n*_engrafted_.

The true value of *n*_final_ is not well established and values of 1 × 10^5^, 2 × 10^5^, 5 × 10^5^, 1 × 10^6^ and 2 × 10^6^ were considered. The lowest value (1 × 10^5^) was chosen as the estimated total HSC population size^24,28^ and the highest value (2 × 10^6^) was selected as the largest value that was computationally feasible. In reality, once the final population size was more than tenfold larger than the starting population, the final population size had little impact on results (Extended Data Fig. 9a). This approach assumes that coalescences are unlikely to occur around the time of GT from ‘steady-state’ hematopoiesis, and therefore that all observed coalescences relate to engraftment. For this reason, the model does not consider parameters such as the steady-state HSC generation time. This is reasonable given (1) the high polyclonality at this young age as evident in the pre-therapy phylogenies (Fig. 1c) and (2) the almost complete absence of coalescences observed in the 5–10 years before sampling in published steady-state hematopoietic phylogenies^28^. Once our estimates had been calculated, we compared these numbers to estimates of engrafting HSPCs from the same individuals based on vector integration site analysis using the R package ‘specpool {vegan}’ (refs. ^71–73^) v.1.15 (Extended Data Fig. 9b).

### Annotation of driver mutations

To identify potential driver mutations, a broad 146-gene list of hematological malignancy-/clonal hematopoiesis-associated genes was compiled from the union of (1) a 54-gene Illumina myeloid panel^74^, (2) the 92-gene list used in a recent study of chemotherapy-associated clonal hematopoiesis^75^, (3) the 95-gene rapid heme panel list adopted by Brigham and Women’s Hospital^37^ and (4) a 32-gene list of genes recently identified as subject to positive selection within the UK Biobank cohort. We looked for missense, truncating or splice variants in these genes, yielding 76 such variants (Supplementary Table 2). These were then manually curated independently by two investigators using the COSMIC database of somatic mutations (https://cancer.sanger.ac.uk/cosmic), the broader literature and, in some cases, variant effect prediction tools such as SIFT and PolyPhen to identify those variants that were potentially pathogenic and those that were of unknown meaning. This curation took place without knowledge of whether the mutation had been found in a pre- or post-therapy sample. Where there was disagreement, discussions were conducted until a consensus was reached.

### Structural variants

Structural variants (SVs) were called with GRIDSS^76^ (v.2.9.4), which was used with default settings. SVs larger than 1 kb in size with QUAL ≥ 250 were included. For SVs smaller than 30 kb, SVs with QUAL ≥ 300 were only included. Furthermore, SVs that had assemblies from both sides of the breakpoint were only considered if they were supported by at least four discordant and two split reads. SVs with imprecise break ends (the distance between the start and end positions was >10 bp) were filtered out. We further filtered out SVs for which the s.d. of the alignment positions at either ends of the discordant read pairs was smaller than five. To remove potential germline SVs and artifacts, we generated the panel of normal by adding in-house normal samples (*n* = 350) to the GRIDSS panel of normal. SVs found in at least three different samples in the panel of normal were removed. SV calls resulting from the GT-integrated vector sequence were filtered by running GRIDSS across the vector sequence only and filtering any called variants from the data. Variants were confirmed by visual inspection and by checking whether they fit the distribution expected based on the SNV-derived phylogenetic tree. Some variants were found in only a subset of colony duplicates, suggesting that they were acquired in vitro. These were all 25–65 kb duplication variants and were excluded from further analysis. The one variant that was found in multiple samples was assigned manually to the appropriate branch on the phylogeny.

### Copy-number alterations

WGS data were analyzed with the software ASCAT^77^ (v.4.2.1), using a matched non-clonally related sample as the ‘normal reference’. Purity was set at 1 and ploidy at 2. Results were manually inspected and alterations that were clearly distinguishable from background noise were tabulated.

### Duplex sequencing

Duplex sequencing was performed with a custom Duplex Sequencing kit (TwinStrand Biosciences). The duplex sequencing in this study was performed on mature myeloid cell samples so data are therefore representative of HSCs actively contributing to the myeloid compartment. For pre-GT samples, the starting cellular material was banked mobilized PB CD34^−^ cells (obtained from the Miltenyi CliniMACS CD34 selection protocol used in the manufacturing of patient investigational medical products). Post-GT BM or PB samples were collected as part of the patient monitoring program. Both types of samples were stained with the following antibodies as recommended by the manufacturer: PerCP-Cy5.5 mouse anti-human CD3 (5 µl per test, clone UCHT1, BD Biosciences, 560835), FITC mouse anti-human CD15 (20 µl per test, clone HI98, BD Biosciences, 555401), APC mouse anti-human CD19 (20 µl per test, clone HIB19, BD Biosciences, 555415) and BV421 mouse anti-human CD56 (5 µl per test, clone NCAM16.2, BD Biosciences, 562751). Myeloid cells were then sorted using either a BD FACSMelody or BD FACSAria instrument and the gating strategy for pre-GT CD3^−^CD19^−^ and post-GT CD15^+^ cells is shown in Supplementary Fig. 2. FlowJo (v.10.8.1) was used for analysis of sorted cell populations. A custom baitset was designed that incorporated 9 of the 12 driver mutations from Fig. 4b (all those that were available from data analyzed at the time of design), along with 40 additional mutations from each of the driver-containing clones. This refers to mutations allocated to the same branch in the phylogeny as the driver mutation. The 40 mutations were arbitrarily selected from the total set of mutations in the clone (usually 400–600) based on (1) the minimum free energy, a metric used to predict whether the probe is likely to fold in on itself, (2) alternate genomic site Blast hits, to minimize off-target capture and (3) % GC content, to minimize issues with poor capture from GC-rich regions. In addition, the baitset incorporated the off-the-shelf TwinStrand AML-29 MRD panel that targets both mutation hotspots and/or full coding sequences in 29 genes recurrently mutated in adult AML^78^. As this panel did not cover all genes commonly mutated in clonal hematopoiesis, nine additional genes were targeted, covering either only hotspots (*SF3B1*, *SRSF2* and *JAK2*) or the full coding sequences (*PPM1D*, *BRCC3*, *CTCF*, *GNB1*, *CHEK2*, *ATM* and *BCOR*).

The 23 DNA samples were shipped to CeGaT in Germany for library preparation and sequencing. Library preparation using various amounts of input genomic DNA (Supplementary Table 3) was performed by ultrasonically shearing the DNA to a mean fragment size of ~300 bp followed by end repair, A-tailing and ligating to TwinStrand DuplexSeq adaptors (TwinStrand Biosciences). After an initial PCR amplification, the desired targets were enriched using the custom baitset of biotinylated oligonucleotides and two tandem captures. Libraries were then sequenced on the Illumina NovaSeq 6000 platform. All 23 samples were multiplexed across a single S4 flow cell. Analysis of initial results suggested that 2 samples had failed, 4 samples had good sequencing results that would not be increased by further sequencing and 17 samples had results that would be further improved by further sequencing. Therefore, libraries from these 17 samples underwent further sequencing on an S2 flow cell. One of the failed samples had further DNA available and underwent repeat library preparation, target enrichment and sequencing. Where samples were re-sequenced, the raw fastq files from the separate sequencing runs were merged before running the TwinStrand analysis pipeline (v.3.20.1), as described by Valentine et al.^79^.

### Assessing clone trajectories

Read counts at all targeted mutation sites (those found in the clone WGS) were assessed from the final consensus bam files using alleleCounter (v.4.3.0; https://github.com/cancerit/alleleCount). To adjust for the hemizygous nature of mutations on the XY chromosomes in males (<5% of targeted mutations), the total read count was multiplied by two at these loci. The average VAF across clone mutations was then calculated at each time point for the individual in whom the driver mutation was originally called by summing the variant counts and total read counts across all clone mutations. The 95% confidence interval was calculated using the base R function ‘binom.test’. To calculate whether the clone had significantly increased in size the aggregated read counts at the final post-therapy time point were compared to the aggregated read counts at the pre-therapy time point using Fisher’s exact test (as implemented in the R function ‘fisher.test’ from the package ‘stats’). The VAF of the driver mutation itself was assessed by looking at the read counts of the driver mutation alone at each time point from the same individual in whom the driver mutation was originally detected.

The additional clone mutations may have been acquired before or after the driver mutation itself. Those acquired before the driver are true passenger mutations, where all cells with the driver mutation also have the passenger mutation, and therefore the VAF of the passenger mutation is always greater than or equal to the VAF of the driver mutation itself. Clone mutations acquired after the driver mutation are in fact subclonal to the driver. The VAF of these mutations may be much lower than the driver mutation itself. In each case it is unknown how many of the selected clone mutations are true passengers or are subclonal. This depends primarily on the timing of driver acquisition: if acquired later, there will have been more acquired passenger mutations and fewer subclonal mutations, whereas if acquired early the reverse will be true. As long as some of the additional clone mutations are true passengers, their inclusion in the sequencing data will increase the sensitivity for the driver clone. Assuming that the driver mutation is equally likely to be acquired at any point in the lifespan of the clone, three-quarters of driver clones will have at least ten true passengers among the 40 sequenced clone mutations. Given our mean sequencing depth of ~12,000x, this would give a combined coverage of ≥132,000× across the ten true passengers and the driver itself. With this coverage one has a >95% chance of detecting a driver clone with a frequency of at least 1 in 22,000 cells (binomial test, assuming the VAF is half of the driver clone frequency due to the heterozygous nature of the acquired variants). Given the inclusion of an unknown number of subclonal variants, the average clone VAF may be considerably lower than the VAF of the driver itself; however, the trajectory of the average clone VAF through time should still be a useful measure of the driver trajectory.

### Retrospective mutation timing from mutation VAFs

In principle, mutations acquired within a single clone will always have a VAF that is equal to or lower than the VAF of mutations previously acquired in that same clone. Mutations detected within a single colony are evidently all within the same clone. Theoretically therefore, if one could know precisely the VAF of all these mutations in a bulk population, one could determine their order of acquisition. This idea has been used to determine the order of mutation acquisition in malignancies^40^.

We used this same principle to establish the timing of driver mutation acquisition in our clones. For each clone we had the bulk sequencing results of the driver mutation itself and 40 passenger mutations (out of a total of 400–600 total mutations in the clone). Given that we have the VAFs of each of the 41 mutations, these can simply be ordered from highest to lowest to obtain a rank for the driver mutation; however, we have to account for uncertainty in the VAFs due to the random binomial sampling of variant/wild-type alleles that make up the read counts from which VAFs are calculated. Therefore, we bootstrapped the read counts of each clone mutation (10,000 bootstraps) and for each bootstrap, calculated the rank *r*_*i*_ of the driver mutation VAF, so that if the driver mutation had the highest VAF it would be ranked first (*r*_*i*_ = 1) and if it had the lowest VAF it would be ranked last (*r*_*i*_ = 41).

However, the rank of the driver mutation among the 40 randomly selected passenger mutations may not accurately reflect its rank among the full set of mutations on the branch. To account for the uncertainty in the distribution of the 41 sequenced mutations among the total set of mutations on the branch, we converted each of the 10,000 initial bootstrap ranks *r*_*i*_ (which were out of 41), to a final rank *r*_*f*_ (out of the full set of 400–600 branch mutations). We did this by randomly selecting 41 numbers from the set {1…*n*_*mut*_} (representing the true ranks of all branch mutations), sorting these in ascending order and finding the number that was in position *r*_*i*_ of the selected number set, giving the final *r*_*f*_.

To convert these ‘ranks’ to an actual time, two further steps were involved. First, the mutation-based tree was converted to a time-based tree using the algorithm rtreefit (v.1.0.1; https://github.com/NickWilliamsSanger/rtreefit)^29^. This assumes a constant mutation acquisition rate after development. This gives a tree where all branch points (representing historic cell divisions) have estimated ages at which they occurred. The true time of driver mutation acquisition corresponding to each bootstrapped rank *r*_*f*_ was then taken as:

$${A}_{{DRIVER}}={A}_{P}+\left(\frac{{r}_{f}}{{n}_{{mut}}}\times \left({A}_{D}-{A}_{P}\right)\right)\,$$

Where *A*_*P*_ is the age of the branch point at the top of the branch (estimated from the time-based tree), *A*_*D*_ is the age of the branch point at the bottom of the branch (also estimated from the time-based tree), *r*_*f*_ is the rank of the driver mutation among all branch mutations and *n*_mut_ is the total number of mutations on the branch. The distribution of *A*_DRIVER_ values obtained from each of the 10,000 bootstraps is shown in Extended Data Fig. 10c. While not all sources of uncertainty are fully accounted for, we believe our method gives a useful estimate of the age of driver mutation acquisition.

### De novo mutation calling from duplex sequencing data

Sequencing data were processed using the TwinStrand analysis pipeline (v.3.20.1) hosted on the DNAnexus platform, which provides bioinformatic facilities^79^. Standard filtering was applied to remove artifacts introduced by end repair and at areas of microsatellite instability. A minimum threshold of three supporting reads was used to call subclonal variants, unless the same variant was called independently in two separate samples from the same individual. Only SNVs were considered, as indel calling was unreliable in certain repetitive regions of the panel. Variants with VAFs consistent with a germline variant (>0.3) were removed and such variants, at any VAF, were also filtered in samples from other individuals as potentially representing cross contamination. In addition, we conservatively reasoned that given that such variants are tolerated in the germline, they are unlikely to substantially affect function. Variants were considered as most likely to alter function if they were annotated as ‘missense_variant’, ‘stop_gained’, ‘splice_region_variant’ or ‘splice_acceptor_variant’ in ClinVarI. Missense variants that were predicted to have a ‘LOW’ functional impact were removed, leaving only those annotated as ‘MODERATE’ or ‘HIGH’. Once called in ≥1 sample, mutation trajectories were then assessed across time points from that individual using alleleCounter (as described above). Lollipop plots of mutations called in *EZH2* and *DNMT3A* were created using the R function ‘g3Lollipop’ from the package ‘g3viz’ (https://github.com/G3viz/g3viz).

Trajectories of variants unlikely to affect cell function were used as a control. These variants were called in exactly the same way as described above, except that only variants annotated as ‘synonymous_variant’, ‘intron_variant’ or ‘upstream_gene_variant’ were included and there was no requirement for any functional impact. Variants at the same sites as those found in the WGS clones were also excluded, as these were passenger mutations of driver variants. When considering the total burden of mutations within a particular gene (Fig. 4e) and the fold change of that burden (Extended Data Fig. 10e), the confidence intervals were calculated using a custom Bayesian inference algorithm (available at https://github.com/mspencerchapman/Gene_therapy).

### Reporting summary

Further information on research design is available in the Nature Portfolio Reporting Summary linked to this article.

## Online content

Any methods, additional references, Nature Portfolio reporting summaries, source data, extended data, supplementary information, acknowledgements, peer review information; details of author contributions and competing interests; and statements of data and code availability are available at 10.1038/s41591-023-02636-6.

### Supplementary information

Supplementary Information Supplementary Figs. 1 and 2 and Tables 1–5.

Reporting summary

## Data Availability

WGS data have been deposited in the European Genome-Phenone Archive (EGA) under accession no. EGAD00001010913 (EGAS00001004620) and targeted sequencing data have been deposited under accession no. EGAD00001010914 (EGAS00001007253). Data from the EGA are accessible for research use only to all bona fide researchers, as assessed by the Data Access Committee (https://www.ebi.ac.uk/ega/about/access). Data can be accessed by registering for an EGA account and contacting the Data Access Committee.

## Code availability

Analysis code, together with extensive derived datasets, is freely available at https://github.com/mspencerchapman/Clonal_selection_after_gene_therapy with some larger elements of the data available on Mendeley Data (10.17632/m7nz2jk8wb.1).
