# Supplementary material for: Clonal selection of hematopoietic stem cells after gene therapy for sickle cell disease
Source: Nat Med. 2023 Nov 16;29(12):3175–83. doi: 10.1038/s41591-023-02636-6 (PMC10719109; doi:10.1038/s41591-023-02636-6)
Supplement: Supplementary file 1 — Supplementary Figs. 1 and 2 and Tables 1–5. [file 41591_2023_2636_MOESM1_ESM.pdf]

nature medicine

Article https://doi.org/10.1038/s41591-023-02636-6

Clonal selection of hematopoietic stem cells after gene therapy for sickle cell disease

In the format provided by the authors and unedited

SUPPLEMENTARY FIGURES AND TABLES

Figure S1. Recognition of colony duplicates. **a,** 96-profile mutation signature of the extracted 'Bone marrow signature' and *in vitro* signature used for fitting mutation profiles from private branches of potential colony duplicates. **b,** Stacked bar plot showing the absolute number of mutations assigned to the 'HSC signature' and *in vitro* signature for all private mutations from potential colony duplicates. These are divided by whether the potential duplicate set were considered "Confident duplicates", "Not all duplicates" or "Unclear status" based on their relative positions in the 96-well plates. Most "Confident duplicates" samples had almost all mutations attributed to the *in vitro* signature. Almost all mutations in the "Not all duplicates" set were attributed to the 'HSPC signature'. Empirical decisions for the "Unclear status" sample sets were then made based on the signature attribution and phylogenetic appearances. The square brackets indicate sample pairs with (1) more private mutations than other potential duplicates, and (2) high numbers of mutations attributed to the 'Bone marrow signature'. These were retained in the data as samples with a close *in vivo* relationship, but that were from different colonies.

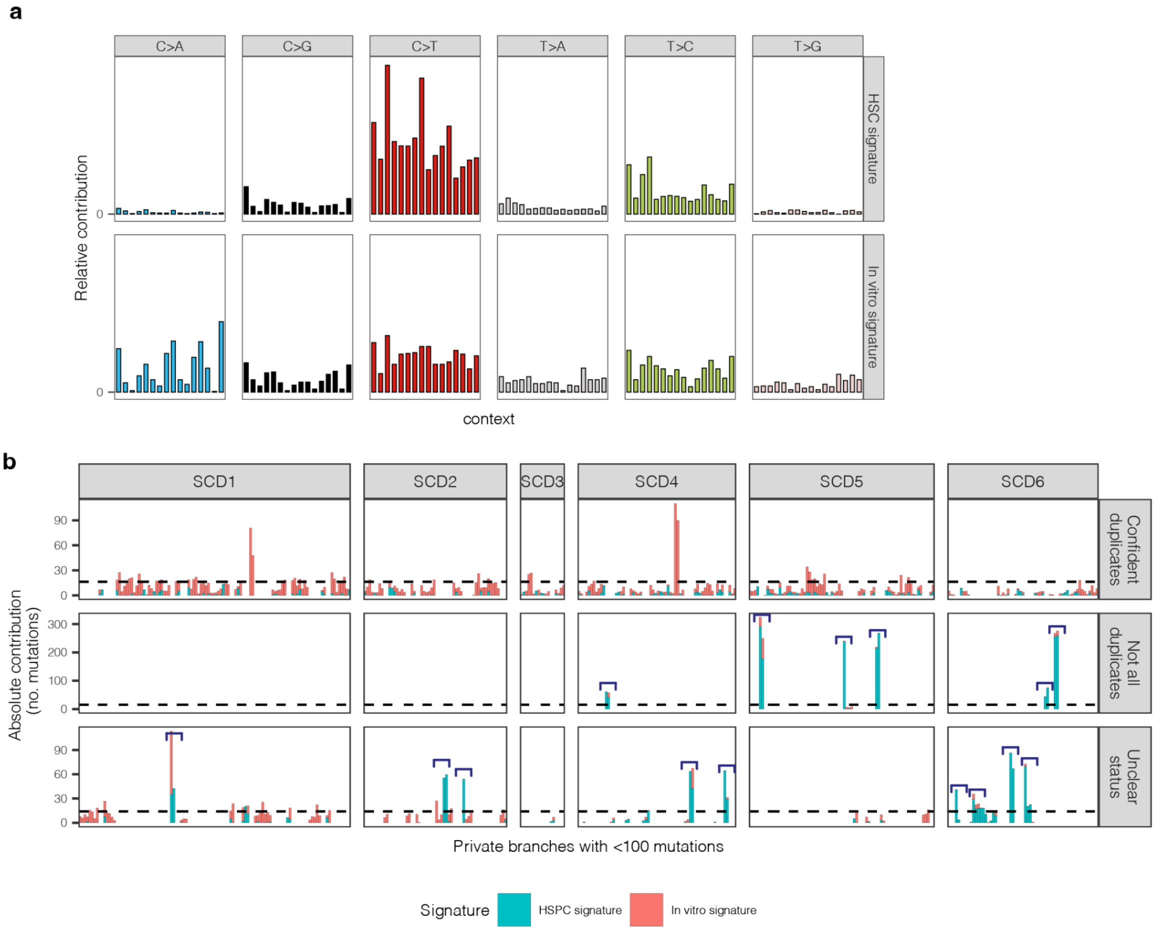

Figure S2. FACS sorting strategy for myeloid cell populations. **a,** Sorting of bulk CD3$^-$CD19$^-$ myeloid cells from pre-GT mobilised PB samples. **b,** Sorting of bulk CD15$^+$ myeloid cells from post-GT PB or BM samples. Cells were stained with the panel of antibodies as indicated in the Online Methods and then cell populations of interest were sorted as bulk populations. Image created with FlowJo v10.

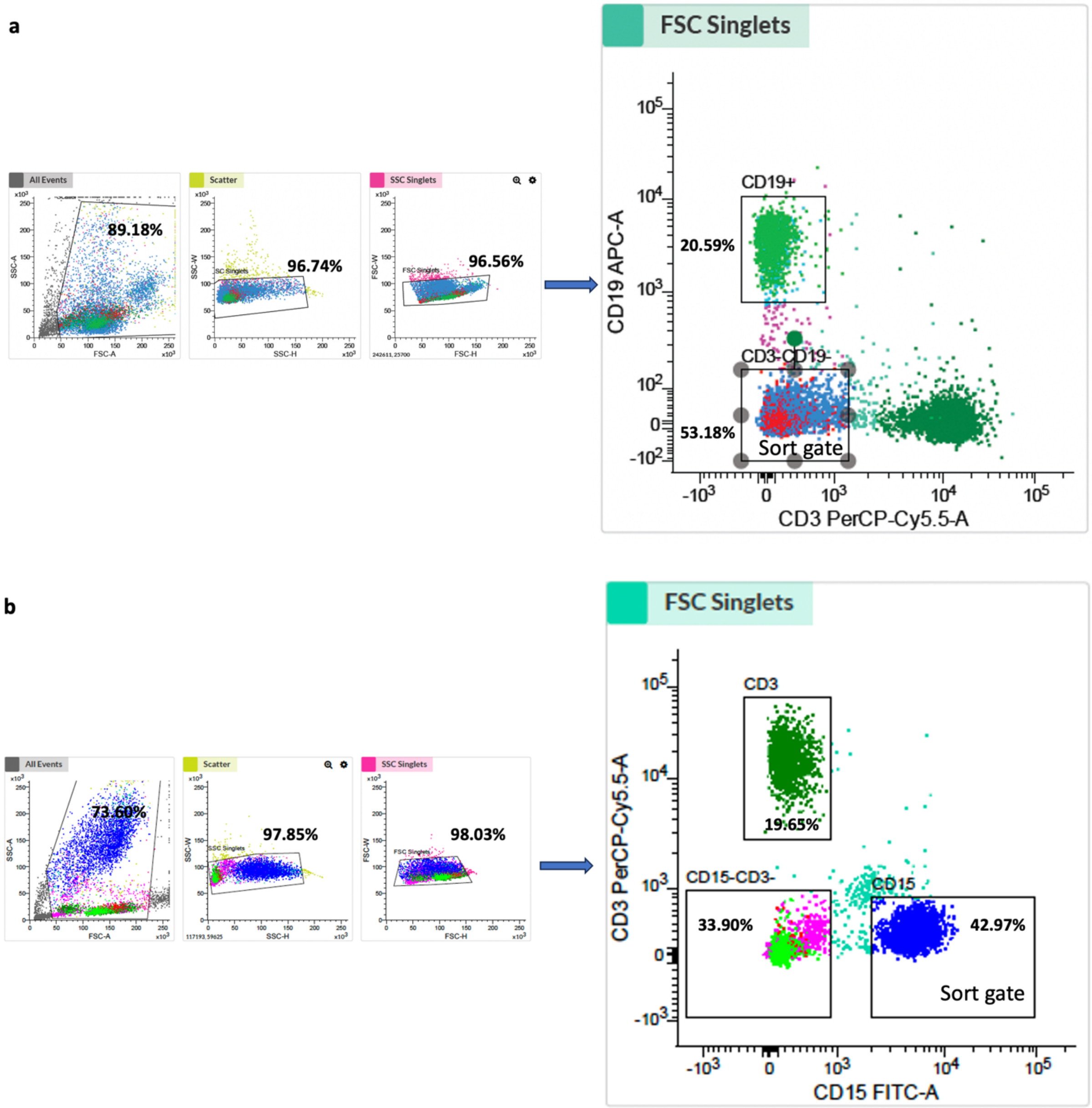

Table S1. Breakdown of cell source and numbers of colonies included in the study.

Patient ID	Cell Source	Timepoint	Sequencing Depth (×)	No. Colonies Sequenced and passing QC
SCD1	PB-MNCs, pre-transduction	Pre-gene therapy	14.6	81
	Drug Product	Pre-gene therapy	13.0	130
	PB-MNCs	24 month follow-up	12.8	143
SCD2	PB-MNCs	Pre-gene therapy	13.6	157
	Drug Product	Pre-gene therapy	11.3	81
	PB-MNCs	21 month follow-up	15.5	74
SCD3	PB-MNCs	Pre-gene therapy	13.5	144
	CD34-enriched BM-MNCs	24 month follow-up	12.4	143
SCD4	*PB-MNCs*	Pre-gene therapy	12.9	266
	CD34-enriched BM-MNCs	24 month follow-up	13.4	154
	PB-MNCs	30 month follow-up	11.8	129
	PB-MNCs	36 month follow-up	13.1	138
SCD5	BM-MNCs	Pre-gene therapy	13.1	155
	PB-MNCs	12 month follow-up	13.8	84
	PB-MNCs	21 month follow-up	12.2	146
	CD34-enriched BM-MNCs	24 month follow-up	12.8	62
SCD6	CD34-enriched BM-MNCs	Pre-gene therapy	12.0	147
	CD34-enriched BM-MNCs	24 month follow-up	10.8	208
	PB-MNCs	30 month follow-up	13.2	150

Table S2. Mutations occurring in potential driver genes, including decisions on variant pathogenicity.

ID	Chrom	Position (GRCh37)	Ref	Mut	Gene	Protein	Gene type	Interpretation
SCD1	2	29449853	C	T	*ALK*	ALK p.S1001N	Oncogene; fusion	VUS
SCD1	7	101918598	C	T	*CUX1*	CUX1 p.R511C	Oncogene; TSG	VUS
SCD1	4	153258959	T	C	*FBXW7*	FBXW7 p.K286E	TSG	VUS
SCD1	20	57429995	C	T	*GNAS*	GNAS p.R559C	Oncogene	VUS
SCD1	21	36252866	G	C	*RUNX1*	RUNX1 p.R166G	Oncogene; TSG; fusion	Potentially Pathogenic
SCD2	11	118374880	T	C	*KMT2A*	KMT2A p.I2758T	Oncogene; fusion	VUS
SCD2	1	120480497	A	G	*NOTCH2*	NOTCH2 p.I1107T	Oncogene; TSG	VUS
SCD2	18	42532882	G	A	*SETBP1*	SETBP1 p.A1193T	Oncogene; fusion	VUS
SCD2	10	112360267	A	G	*SMC3*	SMC3 p.N833S	TSG	VUS
SCD2	4	1957778	T	C	*WHSC1*	WHSC1 p.I915T	Oncogene; fusion	VUS
SCD3	X	129156941	G	A	*BCORL1*	BCORL1 p.R1226H	TSG	VUS
SCD3	X	140984890	A	C	*MAGEC3*	MAGEC3 p.E449A	NA	VUS
SCD3	4	55133754	C	A	*PDGFRA*	PDGFRA p.Q323K	NA	VUS
SCD3	12	111856557	A	G	*SH2B3*	SH2B3 p.Y203C	NA	VUS
SCD3	17	40359611	T	C	*STAT5B*	STAT5B p.K681R	Oncogene; TSG; fusion	VUS
SCD3	4	106196361	C	A	*TET2*	TET2 p.S1565Y	TSG	Potentially Pathogenic
SCD4	3	105422906	C	T	*CBLB*	CBLB p.V507M	TSG	VUS
SCD4	9	21971028	C	T	*CDKN2A*	CDKN2A p.W110*	TSG	Potentially Pathogenic
SCD4	2	25464570	A	C	*DNMT3A*	DNMT3A p.L648R	TSG	Potentially Pathogenic
SCD4	20	31388105	G	A	*DNMT3B*	DNMT3B p.?	TSG	VUS
SCD4	20	31390263	C	T	*DNMT3B*	DNMT3B p.P740S	TSG	VUS
SCD4	7	55221804	G	A	*EGFR*	EGFR p.G283D	Oncogene	VUS
SCD4	22	41489009	A	G	*EP300*	EP300 p.M1V	TSG; fusion	VUS
SCD4	22	41564575	G	A	*EP300*	EP300 p.V1333M	TSG; fusion	VUS
SCD4	7	148508719	C	T	*EZH2*	EZH2 p.E649K	TSG; oncogene	Potentially Pathogenic
SCD4	3	128204837	C	T	*GATA2*	GATA2 p.A202T	Oncogene	VUS
SCD4	15	90631620	C	T	*IDH2*	IDH2 p.V217M	Oncogene	VUS
SCD4	5	35873751	G	A	*IL7R*	IL7R p.?	NA	VUS
SCD4	4	55573378	A	T	*KIT*	KIT p.Q347L	Oncogene	VUS
SCD4	11	118374597	G	A	*KMT2A*	KMT2A p.A2664T	Oncogene; fusion	VUS

SCD4	15	42003129	A	G	*MGA*	MGA p.H889R	TSG	VUS
SCD4	9	139417482	G	C	*NOTCH1*	NOTCH1 p.L188V	TSG	VUS
SCD4	17	58740749	C	T	*PPM1D*	PPM1D p.R552*	TSG	Potentially Pathogenic
SCD4	11	116741090	C	T	*SIK3*	SIK3 p.E531K	TSG	VUS
SCD4	10	112362733	G	A	*SMC3*	SMC3 p.D1150N	TSG	VUS
SCD4	X	123227894	A	T	*STAG2*	STAG2 p.D1202V	TSG	VUS
SCD4	17	40462572	C	T	*STAT5A*	STAT5A p.T757I	Oncogene; TSG; fusion	VUS
SCD4	5	1294576	C	T	*TERT*	TERT p.R142H	Oncogene	VUS
SCD4	17	7577120	C	T	*TP53*	TP53 p.R273H	TSG	Potentially Pathogenic
SCD5	2	29754779	A	C	*ALK*	ALK p.?	Oncogene; fusion	VUS
SCD5	2	29451909	T	TA	*ALK*	ALK p.N886fs*1	Oncogene; fusion	VUS
SCD5	X	76845307	T	C	*ATRX*	ATRX p.K2072E	TSG	VUS
SCD5	19	45285631	G	A	*CBLC*	CBLC p.W221*	TSG	Potentially Pathogenic
SCD5	7	55273279	C	T	*EGFR*	EGFR p.A1201V	Oncogene	VUS
SCD5	7	148507436	T	A	*EZH2*	EZH2 p.N673I	TSG; oncogene	Potentially Pathogenic
SCD5	10	123260396	C	T	*FGFR2*	FGFR2 p.G503E	Oncogene; fusion	VUS
SCD5	15	42003341	G	C	*MGA*	MGA p.D960H	TSG	VUS
SCD5	17	29661984	A	C	*NF1*	NF1 p.M1981L	Oncogene	VUS
SCD5	5	131931342	G	A	*RAD50*	RAD50 p.V683I	TSG	VUS
SCD5	18	42531451	A	C	*SETBP1*	SETBP1 p.S716R	Oncogene; fusion	VUS
SCD5	2	198266566	A	G	*SF3B1*	SF3B1 p.M757T	Oncogene	VUS
SCD5	11	116741090	C	A	*SIK3*	SIK3 p.E531*	TSG	Potentially Pathogenic
SCD5	11	116767923	T	G	*SIK3*	SIK3 p.K185Q	TSG	VUS
SCD5	10	112361838	C	T	*SMC3*	SMC3 p.R1003C	TSG	VUS
SCD5	4	1920238	G	A	*WHSC1*	WHSC1 p.R433K	Oncogene; fusion	VUS
SCD6	12	46245535	T	C	*ARID2*	ARID2 p.V1210A	TSG	VUS
SCD6	16	67655479	C	G	*CTCF*	CTCF p.R448G	TSG	VUS
SCD6	7	55211097	G	A	*EGFR*	EGFR p.E114K	Oncogene	VUS
SCD6	7	55225427	C	T	*EGFR*	EGFR p.R427C	Oncogene	VUS
SCD6	7	55272969	C	G	*EGFR*	EGFR p.P1098A	Oncogene	VUS
SCD6	7	148512045	G	A	*EZH2*	EZH2 p.Q545*	TSG; oncogene	Potentially Pathogenic
SCD6	11	118373953	T	G	*KMT2A*	KMT2A p.F2449C	Oncogene; fusion	VUS
SCD6	15	42042333	AC	A	*MGA*	MGA p.H2177fs*2	TSG	Potentially Pathogenic

SCD6	8	128752788	G	A	*MYC*	MYC p.V317I	Oncogene	VUS
SCD6	17	29557392	G	A	*NF1*	NF1 p.M1035I	Oncogene	VUS
SCD6	4	55138597	A	T	*PDGFRA*	PDGFRA p.H425L	NA	VUS
SCD6	6	79650865	G	C	*PHIP*	PHIP p.P1671A	NA	VUS
SCD6	6	79787767	C	T	*PHIP*	PHIP p.G7S	NA	VUS
SCD6	16	81953097	G	A	*PLCG2*	PLCG2 p.G688D	NA	VUS
SCD6	17	1554232	T	C	*PRPF8*	PRPF8 p.N2291S	TSG	VUS
SCD6	18	42281342	C	T	*SETBP1*	SETBP1 p.R11W	Oncogene; fusion	VUS
SCD6	18	42530173	G	A	*SETBP1*	SETBP1 p.A290T	Oncogene; fusion	VUS
SCD6	18	42643548	C	T	*SETBP1*	SETBP1 p.A1559V	Oncogene; fusion	VUS
SCD6	14	75231066	G	A	*YLPM1*	YLPM1 p.?	NA	VUS
SCD6	6	43316255	C	T	*ZNF318*	ZNF318 p.R960Q	TSG	VUS
SCD6	6	43320199	C	G	*ZNF318*	ZNF318 p.E896Q	TSG	VUS

VUS = variant of uncertain significance; TSG = tumor suppressor gene; NA = not applicable

Table S3. Bulk myeloid samples used for targeted duplex sequencing.

ID	Time point (months)	Source	Sorting strategy	Input DNA (ng)
SCD1	0	mPB	CD34-CD3-CD19-	1250
SCD1	18	PB	CD15+	371
SCD1	24	BM	CD15+	441
SCD2	0	mPB	CD34-CD3-CD19-	1250
SCD2	6	BM	CD15+	180.25
SCD2	12	PB	CD15+	1250
SCD2	18	PB	CD15+	374.5
SCD2	24	PB	CD15+	298.2
SCD3	0	mPB	CD34-CD3-CD19-	665
SCD3	12	PB	CD15+	287.7
SCD3	24	BM	CD15+	284.55
SCD3	30	PB	CD15+	371
SCD4	0	mPB	CD34-CD3-CD19-	651
SCD4	30	PB	CD15+	274.56
SCD4	42	PB	CD15+	444.5
SCD5	0	mPB	CD34-CD3-CD19-	1250
SCD5	24	BM	CD15+	224
SCD5	30	PB	CD15+	288.75
SCD5	36	PB	CD15+	640.5
SCD6	0	mPB	CD34-CD3-CD19-	1250
SCD6	24	BM	CD15+	271.6
SCD6	30	PB	CD15+	546
SCD6	36	PB	CD15+	925

PB = Peripheral blood. BM = Bone marrow. mPB = mobilized Peripheral Blood.

Table S4. Genes covered or partially covered by the targeted duplex sequencing panel.

SF3B1
SRSF2
PPM1D
BRCC3
JAK2
CTCF
GNB1
CHEK2
ATM
BCOR
NRAS
BRINP3
DNMT3A
IDH1
GATA2
KIT
TET2
NPM1
EZH2
RAD21
HNRNPK
PTEN
SMC3
WT1
MLL-X (*KMT2A-X*)
CBL
KRAS
PTPN11
FLT3
IDH2
MYH11-CBFB
TP53
ASXL1
RUNX1
U2AF1
SMC1A
STAG2
PHF6
CEBPA

Table S5. Mutations occurring in potential driver genes as detected by duplex sequencing, including decisions on variant pathogenicity.

ID	Chrom	Position (GRCh38)	Ref	Mut	Gene	Protein	Gene type	Interpretation
SCD1	chr11	108250844	C	T	*ATM*	p.Thr460Met	TSG	VUS
SCD2	chr11	108250981	G	A	*ATM*	p.Gly506Ser	TSG	VUS
SCD3	chrX	40074322	G	C	*BCOR*	p.Arg342Gly	TSG; Fusion	VUS
SCD2	chrX	40063806	G	A	*BCOR*	p.Arg1217*	TSG; Fusion	Potentially Pathogenic
SCD2	chrX	40073385	G	A	*BCOR*	p.Pro654Leu	TSG; Fusion	VUS
SCD2	chr1	190098048	C	T	*BRINP3*	p.Met757Ile	TSG	VUS
SCD6	chr1	190098133	C	T	*BRINP3*	p.Arg729His	TSG	VUS
SCD6	chr1	190098730	C	T	*BRINP3*	p.Arg530His	TSG	VUS
SCD3	chr22	28694072	C	T	*CHEK2*	p.Arg517His	TSG	VUS
SCD1	chr22	28695852	T	C	*CHEK2*	p.Lys416Glu	TSG	Potentially Pathogenic
SCD5	chr2	25235726	A	G	*DNMT3A*	p.Trp860Arg	TSG	Potentially Pathogenic
SCD5	chr2	25240322	C	G	*DNMT3A*	p.Asp768His	TSG	Potentially Pathogenic
SCD2	chr2	25241570	G	C	*DNMT3A*	p.Gln692Glu	TSG	Potentially Pathogenic
SCD4	chr2	25241564	G	A	*DNMT3A*	p.His694Tyr	TSG	Potentially Pathogenic
SCD4	chr2	25244564	A	G	*DNMT3A*	p.Met548Thr	TSG	Potentially Pathogenic
SCD6	chr2	25240360	A	G	*DNMT3A*	p.Phe755Ser	TSG	Potentially Pathogenic
SCD6	chr2	25240691	T	G	*DNMT3A*	p.Ser708Arg	TSG	Potentially Pathogenic
SCD6	chr2	25244604	A	G	*DNMT3A*	p.Ser535Pro	TSG	Potentially Pathogenic
SCD6	chr2	25239166	G	A	*DNMT3A*	p.Ala791Val	TSG	Potentially Pathogenic
SCD6	chr2	25243924	A	G	*DNMT3A*	p.Leu637Pro	TSG	Potentially Pathogenic
SCD6	chr2	25244321	C	T	*DNMT3A*	p.Cys562Tyr	TSG	Potentially Pathogenic
SCD5	chr7	148810344	T	A	*EZH2*	p.Asn673Ile	TSG; Oncogene	Potentially Pathogenic

SCD2	chr7	148810383	C	A	*EZH2*	p.Gly660Val	TSG; Oncogene	Potentially Pathogenic
SCD2	chr7	148826583	A	T	*EZH2*	p.Cys260Ser	TSG; Oncogene	Potentially Pathogenic
SCD4	chr7	148826504	C	T	*EZH2*	p.Cys286Tyr	TSG; Oncogene	Potentially Pathogenic
SCD4	chr7	148811627	C	T	*EZH2*	p.Glu649Lys	TSG; Oncogene	Potentially Pathogenic
SCD6	chr7	148826570	A	C	*EZH2*	p.Ile264Arg	TSG; Oncogene	Potentially Pathogenic
SCD6	chr7	148810374	T	C	*EZH2*	p.Tyr663Cys	TSG; Oncogene	Potentially Pathogenic
SCD4	chr1	1804439	C	G	*GNB1*	p.Arg137Pro	Oncogene	VUS
SCD6	chr1	1789203	G	A	*GNB1*	p.Arg256Cys	Oncogene	VUS
SCD1	chr9	83972891	C	T	*HNRNPK*	p.Asp200Asn	TSG	Potentially Pathogenic
SCD5	chr9	83970251	A	C	*HNRNPK*	p.Asp424Glu	TSG	Potentially Pathogenic
SCD1	chrX	134393949	G	A	*PHF6*	p.Glu139Lys	TSG	Potentially Pathogenic
SCD2	chrX	134413560	G	A	*PHF6*	p.Arg163His	TSG	Potentially Pathogenic
SCD6	chr17	60623733	G	A	*PPM1D*	p.Glu229Lys	Oncogene	Potentially Pathogenic
SCD6	chr17	60663403	C	T	*PPM1D*	p.Arg557*	Oncogene	Potentially Pathogenic
SCD1	chr8	116847581	C	A	*RAD21*	p.Lys605Asn	TSG; Oncogene	Potentially Pathogenic
SCD2	chr8	116854333	T	C	*RAD21*	p.Lys358Arg	TSG; Oncogene	Potentially Pathogenic
SCD3	chr21	34799450	A	G	*RUNX1*	p.Ile273Thr	TSG	Potentially Pathogenic
SCD1	chr21	34880682	G	C	*RUNX1*	p.Thr128Ser	TSG	Potentially Pathogenic
SCD5	chr21	34880568	C	T	*RUNX1*	p.Arg166Gln	TSG	Potentially Pathogenic
SCD3	chr11	116870374	C	A	*SIK3*	p.Glu589*	Unknown	Potentially Pathogenic
SCD4	chrX	53396510	C	A	*SMC1A*	p.Glu890Asp	TSG	Potentially Pathogenic
SCD6	chr10	110602143	C	T	*SMC3*	p.Arg1024Trp	TSG	Potentially Pathogenic
SCD3	chrX	124086638	C	A	*STAG2*	p.Leu1049Ile	TSG	Potentially Pathogenic
SCD3	chr4	105269640	C	T	*TET2*	p.Arg1359Cys	TSG	Potentially Pathogenic

SCD6	chr4	105236198	T	G	*TET2*	p.Asn752Lys	TSG	VUS
SCD2	chr17	7673728	C	A	*TP53*	p.Glu298*	TSG	Potentially Pathogenic
SCD6	chr17	7673803	G	C	*TP53*	p.Arg273Gly	TSG	Potentially Pathogenic

VUS = variant of uncertain significance; TSG = tumor suppressor gene; NA = not applicable
