# Supplementary material for: Clonal selection of hematopoietic stem cells after gene therapy for sickle cell disease
Source: Nat Med. 2023 Nov 16;29(12):3175–83. doi: 10.1038/s41591-023-02636-6 (PMC10719109; doi:10.1038/s41591-023-02636-6)
Supplement: Supplementary file 2 — Reporting summary [file 41591_2023_2636_MOESM2_ESM.pdf]

nature portfolio

Corresponding author(s): David Williams, Peter Campbell, David Kent

Last updated by author(s): 27 September 2023

Reporting Summary

Nature Portfolio wishes to improve the reproducibility of the work that we publish. This form provides structure for consistency and transparency in reporting. For further information on Nature Portfolio policies, see our Editorial Policies and the Editorial Policy Checklist.

Please do not complete any field with "not applicable" or n/a. Refer to the help text for what text to use if an item is not relevant to your study.
For final submission: please carefully check your responses for accuracy; you will not be able to make changes later.

Statistics

For all statistical analyses, confirm that the following items are present in the figure legend, table legend, main text, or Methods section.

n/a	Confirmed	
☐	☒	The exact sample size (*n*) for each experimental group/condition, given as a discrete number and unit of measurement
☐	☒	A statement on whether measurements were taken from distinct samples or whether the same sample was measured repeatedly
☐	☒	The statistical test(s) used AND whether they are one- or two-sided<br>*Only common tests should be described solely by name; describe more complex techniques in the Methods section.*
☐	☒	A description of all covariates tested
☐	☒	A description of any assumptions or corrections, such as tests of normality and adjustment for multiple comparisons
☐	☒	A full description of the statistical parameters including central tendency (e.g. means) or other basic estimates (e.g. regression coefficient) AND variation (e.g. standard deviation) or associated estimates of uncertainty (e.g. confidence intervals)
☐	☒	For null hypothesis testing, the test statistic (e.g. *F*, *t*, *r*) with confidence intervals, effect sizes, degrees of freedom and *P* value noted<br>*Give P values as exact values whenever suitable.*
☐	☒	For Bayesian analysis, information on the choice of priors and Markov chain Monte Carlo settings
☐	☒	For hierarchical and complex designs, identification of the appropriate level for tests and full reporting of outcomes
☒	☐	Estimates of effect sizes (e.g. Cohen's *d*, Pearson's *r*), indicating how they were calculated

Our web collection on statistics for biologists contains articles on many of the points above.

Software and code

Policy information about availability of computer code

Data collection

No software was used for data collection.

Data analysis

Read alignments were performed using BWA-MEM, version 0.7.17 (https://sourceforge.net/projects/bio-bwa/). Single-nucleotide variants were called using the CaVEMan (cancer variants through expectation maximization) algorithm, version 1.13.14 (https://github.com/cancerit/cgpCaVEManWrapper) and initial filtering performed with SangerLCMFiltering, version 1.03 (https://github.com/MathijsSanders/SangerLCMFiltering). cgpVAF, version 5.6.1, was used to create variant read and depth matrices from the bedfiles of mutations called in any individual sample (https://github.com/cancerit/vafCorrect). Small insertions and deletions were called using the Pindel algorithm, version 3.3.0 (https://github.com/cancerit/cgpPindel). Copy number analysis was performed using ASCAT, version 4.2.1. Structural variants were called by GRI DSS, version 2.9.4 (http://github.com/PapenfussLab/gridss). Estimates of engrafting HSPCs based on vector integration site analysis was done using 'specpool {vegan}, version 1.15. Tree building was performed with MPBoot version 1.1.0 for Linux (http://
www.iqtree.org/mpboot). Conversion of tree branches to a time-based tree was done using the algorithm rtreefit version 1.0.1 (https://github.com/NickWilliamsSanger/rtreefit). Mutation assignment to the tree was performed with the treemut package version 1.1 (https://github.com/ NickWilliamsSanger/treemut). Mutational signatures were extracted using the R package HDP version 0.1.5 (https://
github.com/nicolaroberts/hdp). A linear mixed-effects regression approach in the R package 'nlme' version 3.1 was used to assess increases in mutation acquisition (https://cran.r-project/package=nlme)*In vitro* signatures were defined using the 'fit_to_signatures' function from the R package MutationalPatterns, version 3.14 (http://dio.org/dio:10.18129). The R package 'Ckmeans.1d.dp' version 4.3.4 was used to cluster vector integration site reads to the same locations/chromosomes. TwinStrand data read counts were assessed using alleleCounter version 4.3.0 (https://github.com/cancerit/alleleCount). Sequencing data was processed using the TwinStrand analysis pipeline version 3.20.1. The following open source R packages were used in the analyses presented throughout this paper: data.table (v1.12.8), ggplot2 (v3.3.0), stringr (v1.4.0), seqinr (v3.6-1), tidyr (v1.0.2), dplyr (v0.8.5), plotrix (v3.7-7), phangorn (v2.5.5) , RColorBrewer (v1.1-2), ape (v5.3), phytools (v0.6-99), VGAM (v1.1-2), gridExtra (v2.3), pheatmap (v1.0.12). FlowJo (v10.8.1) was used for analysis of sorted cell populations. Miscellaneous scripts for downstream analysis are available on GitHub (https://github.com/mspencerchapman/Clonal_selection_after_gene_therapy).

For manuscripts utilizing custom algorithms or software that are central to the research but not yet described in published literature, software must be made available to editors and reviewers. We strongly encourage code deposition in a community repository (e.g. GitHub). See the Nature Portfolio guidelines for submitting code & software for further information

Data

Policy information about availability of data

All manuscripts must include a data availability statement. This statement should provide the following information, where applicable:

- Accession codes, unique identifiers, or web links for publicly available datasets
- A description of any restrictions on data availability
- For clinical datasets or third party data, please ensure that the statement adheres to our policy

Sequence data that support the findings of this study have been deposited in the European Genome-Phenome Archive (https://www.ebi.ac.uk/ega/home; accession number to be added). All scripts and some smaller data matrices are available on github (https://github/mspencerchapman/Clonal_selection_after_gene_therapy) with some larger elements of the data available on Mendeley Data (DOI: 10.17632/m7nz2jk8wb.1). hg37 human reference genome has been used in this study.

Research involving human participants, their data, or biological material

Policy information about studies with human participants or human data. See also policy information about sex, gender (identity/presentation), and sexual orientation and race, ethnicity and racism.

Reporting on sex and gender	Information on patient sex is reported in Table 1 of the main text.
Reporting on race, ethnicity, or other socially relevant groupings	Patient ethnicity is not specifically reported in this study but this information is available as part of the clinical trial that they are participating in (NCT03282656).
Population characteristics	Patients ranged from 7-26 years of age and are participating in an ongoing clinical trial (NCT03282656).
Recruitment	Recruitment of patients was done through an active clinical trial (NCT03282656).
Ethics oversight	This work was conducted under the existing ethics for the clinical trial (NCT03282656).

Note that full information on the approval of the study protocol must also be provided in the manuscript.

Field-specific reporting

Please select the one below that is the best fit for your research. If you are not sure, read the appropriate sections before making your selection.

[X] Life sciences [ ] Behavioural & social sciences [ ] Ecological, evolutionary & environmental sciences

For a reference copy of the document with all sections, see nature.com/documents/nr-reporting-summary-flat.pdf

Life sciences study design

All studies must disclose on these points even when the disclosure is negative.

Sample size	We optimized the number of individuals (6) and number of hematopoietic stem cells sequenced per individual (average of 432 cells per individual) to describe the mutation burden and clonal structure of both pre- and post-GT hematopoietic stem cell populations across patients. No power calculations were performed and there was no target effect size.
Data exclusions	Per pre-established criteria, in vitro-scquired mutations, sequencing artefacts and samples with very low sequencing coverage were excluded from downstream analysis. See Supplementary Appendix for details.
Replication	We replicated the experiment on a total of 6 patient sample sets (including samples collected both prior to and following gene therapy). No further experiments have since been performed.
Randomization	This was not relevant to our study as we were interested in looking at pre- and post-gene therapy samples from the same individuals. There were no hematopoietically normal individuals involved and there were no test versus control groups.
Blinding	Blinding was not relevant to this study. There were no intervention/control arms in this study.

Behavioural & social sciences study design

All studies must disclose on these points even when the disclosure is negative.

Study description	
Research sample	
Sampling strategy	
Data collection	
Timing	
Data exclusions	
Non-participation	
Randomization	

Ecological, evolutionary & environmental sciences study design

All studies must disclose on these points even when the disclosure is negative.

Study description

Research sample

Sampling strategy

Data collection

Timing and spatial scale

Data exclusions

Reproducibility

Randomization

Blinding

Did the study involve field work? ☐ Yes ☐ No

Field work, collection and transport

Field conditions

Location

Access & import/export

Disturbance

Reporting for specific materials, systems and methods

We require information from authors about some types of materials, experimental systems and methods used in many studies. Here, indicate whether each material, system or method listed is relevant to your study. If you are not sure if a list item applies to your research, read the appropriate section before selecting a response.

Materials & experimental systems

n/a	Involved in the study	
☐	[X]	Antibodies
[X]	☐	Eukaryotic cell lines
[X]	☐	Palaeontology and archaeology
[X]	☐	Animals and other organisms
[X]	☐	Clinical data
[X]	☐	Dual use research of concern
[X]	☐	Plants

Methods

n/a	Involved in the study	
[X]	☐	ChIP-seq
☐	[X]	Flow cytometry
[X]	☐	MRI-based neuroimaging

Antibodies

Antibodies used

PerCP-Cy5.5 Mouse Anti-Human CD3 (clone UCHT1, BD Biosciences, 560835), FITC Mouse Anti-Human CD15 (clone HI98, BD Biosciences, 555401), APC Mouse Anti-Human CD19 (clone HIB19, BD Biosciences, 555415) and BV421 Mouse Anti-Human CD56 (clone NCAM16.2, BD Biosciences, 562751).

Validation

These were all previously validated commercially available antibodies.
PerCP-Cy5.5 CD3: Validated by supplier with the following notes - species reactivity: human ; application: flow cytometry
FITC CD15: Validated by supplier with the following notes - species reactivity: human ; application: flow cytometry
APC CD19: Validated by supplier with the following notes - species reactivity: human ; application: flow cytometry
BV421 CD56: Validated by supplier with the following notes - species reactivity: human ; application: flow cytometry

Eukaryotic cell lines

Policy information about cell lines and Sex and Gender in Research

Cell line source(s)

Authentication

Mycoplasma contamination

Commonly misidentified lines
(See ICLAC register)

Palaeontology and Archaeology

Specimen provenance

Specimen deposition

Dating methods

☐ Tick this box to confirm that the raw and calibrated dates are available in the paper or in Supplementary Information.

Ethics oversight

Note that full information on the approval of the study protocol must also be provided in the manuscript.

Animals and other research organisms

Policy information about studies involving animals; ARRIVE guidelines recommended for reporting animal research, and Sex and Gender in Research

Laboratory animals

Wild animals

Reporting on sex

Field-collected samples

Ethics oversight

Note that full information on the approval of the study protocol must also be provided in the manuscript.

Clinical data

Policy information about clinical studies
All manuscripts should comply with the ICMJE guidelines for publication of clinical research and a completed CONSORT checklist must be included with all submissions.

Clinical trial registration

Study protocol

Data collection

Outcomes

Dual use research of concern

Policy information about dual use research of concern

Hazards

Could the accidental, deliberate or reckless misuse of agents or technologies generated in the work, or the application of information presented in the manuscript, pose a threat to:

No | Yes

- [ ] [ ] Public health
- [ ] [ ] National security
- [ ] [ ] Crops and/or livestock
- [ ] [ ] Ecosystems
- [ ] [ ] Any other significant area

Experiments of concern

Does the work involve any of these experiments of concern:

No | Yes

- [ ] [ ] Demonstrate how to render a vaccine ineffective
- [ ] [ ] Confer resistance to therapeutically useful antibiotics or antiviral agents
- [ ] [ ] Enhance the virulence of a pathogen or render a nonpathogen virulent
- [ ] [ ] Increase transmissibility of a pathogen
- [ ] [ ] Alter the host range of a pathogen
- [ ] [ ] Enable evasion of diagnostic/detection modalities
- [ ] [ ] Enable the weaponization of a biological agent or toxin
- [ ] [ ] Any other potentially harmful combination of experiments and agents

Plants

Seed stocks

Novel plant genotypes

Authentication

ChIP-seq

Data deposition

- [ ] Confirm that both raw and final processed data have been deposited in a public database such as GEO.
- [ ] Confirm that you have deposited or provided access to graph files (e.g. BED files) for the called peaks.

Data access links
May remain private before publication.

Files in database submission

Genome browser session
(e.g. UCSC)

Methodology

Replicates

Sequencing depth

Antibodies

Peak calling parameters

Data quality

Software

Flow Cytometry

Plots

Confirm that:

[X] The axis labels state the marker and fluorochrome used (e.g. CD4-FITC).

[X] The axis scales are clearly visible. Include numbers along axes only for bottom left plot of group (a 'group' is an analysis of identical markers).

[X] All plots are contour plots with outliers or pseudocolor plots.

[X] A numerical value for number of cells or percentage (with statistics) is provided.

Methodology

Sample preparation	For pre-GT samples, the starting cellular material was banked mobilised PB CD34- cells obtained from the Miltenyi CliniMACS CD34 selection protocol used in the manufacturing of patient investigational medical products. Post-GT BM or PB samples were collected as part of the clinical trial's patient monitoring program. These samples did not undergo a CD34 enrichment step.
Instrument	Samples were sorted on either a BD FACSMelody or a BD FACSAria.
Software	No analysis of FACS data is presented in this manuscript. Flowjo v10 was used to generate the gating strategy figure.
Cell population abundance	In pre-GT samples, CD3-CD19- myeloid cells were ~40% of live cells. In post-GT samples, CD15+ myeloid cells made up ~50% of live cells.
Gating strategy	Gating strategies for both pre- and post-GT samples are shown in Figure S2. To summarize:<br>1.SSC-A vs FSC-A showing all events: gate on overall cell population (to exclude dead cells and debris)<br>2.SSC-W vs SSC.H and FSC-W vs. FSC-H showing cell population: gate on singlets (to exclude doublets)<br>3.For pre-GT: CD19 vs. CD3 showing singlets: gate on CD3-CD19- (myeloid cells). For post-GT: CD3 vs CD15 showing singlets: gate on CD15+ (myeloid cells)

[X] Tick this box to confirm that a figure exemplifying the gating strategy is provided in the Supplementary Information.

Magnetic resonance imaging

Experimental design

Design type	
Design specifications	
Behavioral performance measures	

Imaging type(s)	
Field strength	
Sequence & imaging parameters	
Area of acquisition	

Diffusion MRI [ ] Used [ ] Not used

Preprocessing

Preprocessing software	
Normalization	
Normalization template	
Noise and artifact removal	
Volume censoring	

Statistical modeling & inference

Model type and settings	
Effect(s) tested	

Specify type of analysis: [ ] Whole brain [ ] ROI-based [ ] Both

Statistic type for inference

(See Eklund et al. 2016)

Correction

Models & analysis

n/a	Involved in the study
☐	☐ Functional and/or effective connectivity
☐	☐ Graph analysis
☐	☐ Multivariate modeling or predictive analysis

Functional and/or effective connectivity

Graph analysis

Multivariate modeling and predictive analysis

This checklist template is licensed under a Creative Commons Attribution 4.0 International License, which permits use, sharing, adaptation, distribution and reproduction in any medium or format, as long as you give appropriate credit to the original author(s) and the source, provide a link to the Creative Commons license, and indicate if changes were made. The images or other third party material in this article are included in the article's Creative Commons license, unless indicated otherwise in a credit line to the material. If material is not included in the article's Creative Commons license and your intended use is not permitted by statutory regulation or exceeds the permitted use, you will need to obtain permission directly from the copyright holder. To view a copy of this license, visit http://creativecommons.org/licenses/by/4.0/
